# A whispering-gallery scanning microprobe for Raman spectroscopy and imaging

**DOI:** 10.1038/s41377-023-01276-2

**Published:** 2023-10-05

**Authors:** Wenbo Mao, Yihang Li, Xuefeng Jiang, Zhiwen Liu, Lan Yang

**Affiliations:** 1 Department of Electrical and Systems Engineering, Washington University, St Louis, MO 63130 USA; 2 Department of Electrical Engineering, Pennsylvania State University, University Park, PA 16802 USA

**Keywords:** Optical sensors, Raman spectroscopy, Nonlinear optics

## Abstract

Optical whispering-gallery-mode microsensors are a promising platform for many applications, such as biomedical monitoring, magnetic sensing, and vibration detection. However, like many other micro/nanosensors, they cannot simultaneously have two critical properties – ultrahigh sensitivity and large detection area, which are desired for most sensing applications. Here, we report a novel scanning whispering-gallery-mode microprobe optimized for both features and demonstrate enhanced Raman spectroscopy, providing high-specificity information on molecular fingerprints that are important for numerous sensing applications. Combining the superiorities of whispering-gallery modes and nanoplasmonics, the microprobe exhibits a two-orders-of-magnitude sensitivity improvement over traditional plasmonics-only enhancement; this leads to molecular detection demonstrated with stronger target signals but less optical power required than surface-enhanced-Raman-spectroscopy substrates. Furthermore, the scanning microprobe greatly expands the effective detection area and realizes two-dimensional micron-resolution Raman imaging of molecular distribution. The versatile and ultrasensitive scanning microprobe configuration will thus benefit material characterization, chemical imaging, and quantum-enhanced sensing.

## Abstract (web summary)

Conventional surface-enhanced Raman spectroscopy (SERS) techniques are improved by a scannable whispering-gallery-mode (WGM) optical microprobe.

## Graphical abstract

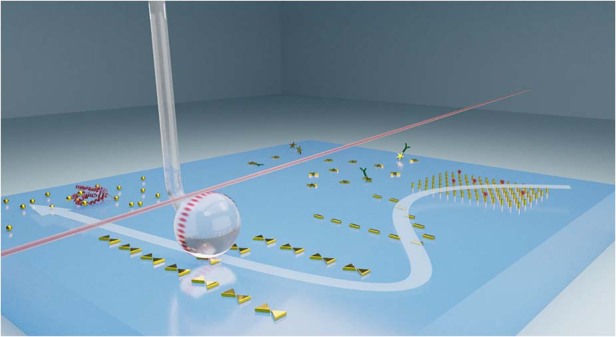

## Introduction

Light-matter interaction plays an essential role in photonic applications such as optical sensing, spectroscopy, lasers, and quantum information technologies. It can be enhanced with optical resonators by temporal accumulation and spatial confinement of light fields. Ultrahigh-quality-factor (*Q*) whispering-gallery-mode (WGM) microresonators, in which light propagates in a circular structure without significant dissipation until millions of roundtrips, are found as a prominent candidate in sensors^1,2^, microlasers^3,4^, and nonlinear optical systems^5^. High-sensitivity WGM sensing is one of the important applications and has been extensively investigated in molecule/particle detection^6–15^, vibration sensing^16,17^, temperature monitoring^18,19^, and optical spectroscopies^20–22^. The long photon lifetime and strong spatial confinement of light in a WGM microsensor, characterized by a high *Q* factor and small mode volume *V*, respectively, enhance the interaction of light with analytes, leading to high sensitivity. To further boost the detection limit, plasmonic nanostructures, such as gold nanorods, are introduced to enhance the light-matter interaction at the surface of WGM microsensors. The detections of a single molecule or atomic ion have been reported in the WGM-nanoplasmonics hybrid systems^10–12,14^.

However, in a conventional setting including a stationary WGM sensor, the effective sensing area is limited as the evanescent field for sensing is distributed within a confined region on the microresonator surface and extends to the surrounding medium at a wavelength scale, requiring targets of interest to enter the probed areas. Likewise, plasmonic nanostructures enhance light-matter interactions by confining optical fields in deep subwavelength volumes^23–26^, and the changes they can effectively detect are only in the nanometer range of the surface.

To address the aforementioned challenges, a novel WGM scanning microprobe is proposed and implemented as a configuration similar to an atomic-force-microscopy (AFM) tip. In this configuration, by leveraging the evanescent field extending to the surrounding medium on a micron scale, our movable WGM sensor can scan and detect targets of interest across a significantly larger effective area. This enhanced mobility expands the WGM sensor’s capability to detect targets beyond the limitations of a conventional setup. In addition, instead of randomly adding plasmonic nanostructures on the surface of a WGM resonator, in this work, high-*Q* WGMs and nanoplasmonics are coupled through scanning controlled by a motorized translation stage with a spatial resolution of 20 nm. The highly controlled optoplasmonic hybrid resonances significantly enhance light-matter interactions for high-sensitivity molecular detection. The WGM microprobe can scan the sample with random plasmonic nanostructures such as commercially available surface-enhanced-Raman-spectroscopy (SERS) substrates. Furthermore, leveraging semiconductor-compatible fabrication technologies, periodic nanostructures can be created to enable phase-matched interactions of the WGM and multiple nanoplasmonic modes to maximize the optoplasmonic-hybrid-enhanced sensitivity. In addition, beyond the previously reported hybrid sensors only monitoring enlarged resonance shift and broadening^10–12,14,15^, we demonstrate sensing with high specificity by introducing Raman spectroscopy to the WGM microprobe. Two-dimensional (2D) hyperspectral imaging with a resolution of ~2 μm is demonstrated by spatially mapping the Raman spectra of target molecules (4-nitrothiophenol, pNTP). An additional two-orders-of-magnitude signal improvement is achieved on top of widely used SERS techniques^25–27^. The proposed WGM microprobe extends the effective sensing area to the whole sample surface. Thus, it provides a versatile platform to study cavity-antenna coupling, enhance nonlinear optical signals, and acquire 2D imaging of samples.

## Results

### Scanning WGM microprobe

**Fig. 1:**
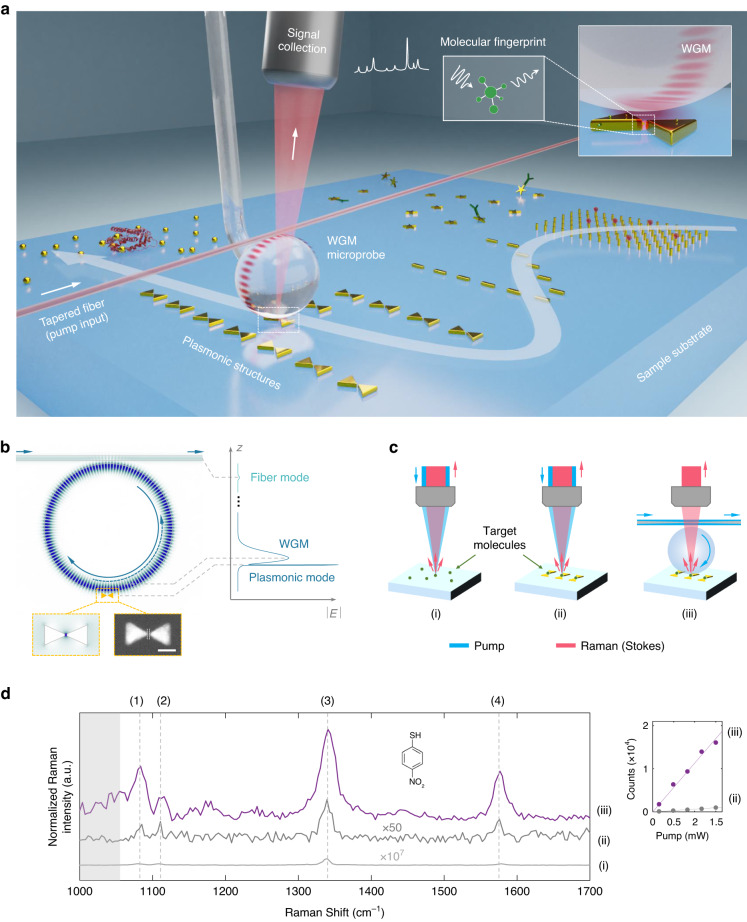
Configuration of a WGM microprobe. **a** Schematic of a scanning WGM microprobe for ultrahigh-sensitivity molecular detection and imaging. The optoplasmonic hybrid resonance is formed by evanescently coupling the WGM microprobe with plasmonic nanostructures on a substrate. The doubly enhanced Raman spectroscopy, benefiting from both high-*Q* WGMs and nanoplasmonics, enables high-sensitivity acquisition of molecular fingerprints. The 2D Raman imaging can be realized by the scannable configuration. **b** Numerical simulation of the optoplasmonic hybrid mode. The amplitude of the electric field $|{E}|$ distributed along the *z*-direction is plotted for the fiber mode, WGM, and plasmonic mode, respectively. The field enhancements are contributed from both the WGM and nanoplasmonics. Bottom inset: simulated electric field (left) and SEM image (right) of a bowtie-shaped nanoantenna. Scale bar: 100 nm. **c** Different configurations to excite Raman scattering. Target molecules (4-nitrothiophenol, pNTP) are (i) pumped by free-space light without enhancement, (ii) pumped by free-space light with nanoplasmonic enhancement, and (iii) pumped by the microprobe with WGM-nanoplasmonics double enhancement. Raman signals are collected by a free-space spectrometer (not shown). **d** Raman spectra corresponding to three configurations in **c**. The input pump power and the number of excited molecules are normalized for comparison among Raman intensities. Inset: the photon counts of the peak (3) proportionally increase with the pump power

The microsphere WGM resonator is mounted on a 3D nano-translation stage through a supporting fiber stem for scanning a sample substrate (Fig. 1a). Using chemical synthesis, self-assembling, or semiconductor-compatible fabrication technologies, the substrate can be patterned with functional structures, such as plasmonic nanobowties, nanospheres, nanostars, nanorods, and nanopillars bonded with analytes to be detected. The optoplasmonic hybrid resonance is formed when the WGM microprobe approaches the substrate and is coupled with the plasmonic nanostructures. The coupling distance is optimized to enhance the light-matter interactions for high-sensitivity molecular detection, and the WGM microprobe is scanned horizontally to extend the sensing area and realize 2D mapping of signals.

The microsphere used in this work has a diameter ~40 μm and an intrinsic *Q* factor ~10^7^. The bowtie-shaped nanoantennas, consisting of two triangles separated by ~15 nm, are exemplified as plasmonic structures on the substrate (Supplementary Section 3). The formed hybrid resonance takes advantage of both temporal accumulation and spatial confinement of light in a nanoscale mode volume. The double enhancements of the optical field, i.e., resulting from high-*Q* WGM and nanoplasmonics, respectively, are shown by the numerical simulation with an optical waveguide input port (Fig. 1b). Such strong enhancement can not only improve the sensitivity of sensors based upon linear optical response, but also facilitate diverse nonlinear optical applications. Raman spectroscopy is used as an example in this work to demonstrate the enhanced light-matter interactions in the WGM microprobe.

### Enhanced Raman spectroscopy

Raman spectroscopy is widely used for identifying and characterizing sample materials by probing fingerprint-like molecular vibrations. However, every million or billion incident photons produce only one Raman photon due to small scattering cross-sections (~10^−30^ cm^2^)^26^. The significant enhancements in the WGM microprobe configuration can be applied to Raman spectroscopy to improve weak Raman signals. The WGM-nanoplasmonics hybrid resonance is efficiently excited through an optical waveguide (tapered fibers). Compared with the focused free-space optical pump in conventional SERS systems, the light accumulation in the high-*Q* WGM microsphere enhances the pump intensity, thereby greatly improving the sensitivity of Raman spectroscopy. The Stokes Raman photons are collected by a long-working-distance objective (NA = 0.55) and detected by a liquid-nitrogen-cooled spectrometer. The detailed experimental setup can be found in Supplementary Section 2.

To evaluate the signal enhancement, we compared the microprobe-based doubly enhanced Raman spectroscopy (iii) with the conventional free-space pumped SERS (ii) and the Raman scattering from a bulk sample without any enhancement (i) (Fig. 1c). The characteristic Raman shifts of target molecules (pNTP), 1080 cm^−1^, 1110 cm^−1^, 1340 cm^−1^, and 1575 cm^−1^, are marked in the spectra of Fig. 1d. The background signals, such as the thermal noise of the spectrometer and the broad Raman scattering of silica materials, are removed by subtracting the spectra without target molecules. The intensity of Raman scattering is evaluated by the height of the peak (3), which originates from the symmetric stretching vibration of the NO_2_ group. The Raman photon counts are found to be proportional to the input pump power, as expected for spontaneous Raman scattering (Fig. 1d, inset). The Raman signal from configuration (iii) is stronger than that from configuration (ii), indicating an additional enhancement factor (EF) 1.1 × 10^2^ realized by WGM microprobe over the free-space pumped SERS, with a total EF of up to 10^8^ (see Methods for details).

To understand the physics of cavity-antenna coupling and the approaches to maximizing the microprobe performance, we derived the enhanced field intensity with the WGM microprobe (Supplementary Section 1),

1 $${\left|{{\boldsymbol{E}}}_{{\boldsymbol{a}}}\right|}^{2}\approx \frac{C}{{V}_{a}\left({\Delta }^{2}+{\Gamma }_{a}^{2}\right)}\cdot \frac{{\kappa }_{{\rm{in}}}}{{\Delta }_{{\rm{in}}}^{2}+{\left({\Gamma }_{c}+\delta {\Gamma }_{c}+{\kappa }_{{\rm{in}}}\right)}^{2}}\cdot {g}_{{ca}}^{2}$$

The capacity of confining light by nanoplasmonics is determined by mode volume ${V}_{a}$, plasmonic dissipation ${\Gamma }_{a}$, and cavity-antenna detuning $\Delta$. The performance of the WGM microprobe is affected by the parameters of the hybrid resonance (detuning of input light ${\Delta }_{{\rm{in}}}$, overall dissipation ${\Gamma }_{c}+\delta {\Gamma }_{c}+{\kappa }_{{\rm{in}}}$, and external coupling strength ${\kappa }_{{\rm{in}}}$) as well as the cavity-antenna coupling strength ${g}_{{ca}}$. The cavity-antenna coupling results in a linewidth broadening $\delta {\Gamma }_{c}$ to the cavity mode (${\Gamma }_{c}$). The coefficient $C$ includes the power of the input light.

**Fig. 2:**
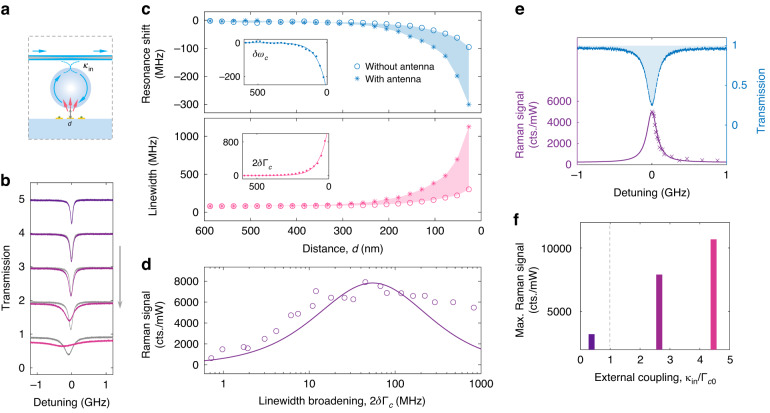
Excitation of Raman scattering through a WGM microprobe. **a** Side-view schematic. The distance between the microsphere cavity and the substrate is ${d}$, and the hybrid mode is excited through the external coupling (${\kappa }_{{\rm{in}}}$) from a tapered fiber. **b** Transmission spectra as coupled to the substrate with (colored) and without (grayed) nanoantennas. The distances ${d}$ from top to bottom are 536 nm, 332 nm, 230 nm, 128 nm, and 26 nm, respectively. **c** Resonance shift and linewidth broadening of the hybrid mode as the distance is varied. Inset: characteristic changes of the hybrid mode ($\delta {\omega }_{c}$ and $\delta {\Gamma }_{c}$) induced by nanoantennas. **d** Variation of Raman signals as the antennas approach the microsphere cavity. **e** Transmission spectrum of a hybrid mode (blue) and Raman signals at different detuning of the pump light (purple). **f** Maximum signals that can be obtained by optimizing the distance ${d}$ at different external coupling strengths ${\kappa }_{{\rm{in}}}$. ${\Gamma }_{c0}$, intrinsic dissipation of the cavity without antennas coupled

We investigated various parameters in Eq. (1) and their effects on Raman signals. The cavity-antenna coupling strength, i.e., the field overlapping between the WGM and nanoplasmonics, influences the excitation efficiency of molecular Raman scattering with the WGM microprobe. To study this effect, the coupling distance $d$ was precisely controlled by a nano-translation stage while the external coupling ${\kappa }_{{\rm{in}}}$ was fixed (Fig. 2a). As the nanoantennas on the substrate approached the WGM cavity, the transmission spectra of the WGM experienced linewidth broadening due to coupling with the metallic-loss-dominated plasmonic modes (Fig. 2b), which indicates the excitation of an optoplasmonic hybrid mode. The resonance shifts and linewidths (Fig. 2c) were extracted from the transmission spectra by Lorentzian curve fitting. The substrate effect was deducted to obtain the resonance shift and linewidth broadening solely induced by nanoantennas, $\delta {\omega }_{c}$ and $\delta {\Gamma }_{c}$ (inset). Their exponential trends, consistent with the spatial decay of WGMs (~780 nm) outside the cavity, confirm that the cavity-antenna coupling of the microprobe is mainly contributed by evanescent field overlapping. The stronger field overlapping improves the excitation efficiency of Raman scattering from target molecules (pNTP) inside plasmonic hotspots; however, at the same time, the increased loss of the optoplasmonic mode could reduce the Raman enhancement. There exists a critical coupling strength for optimal Raman signal generation. As shown in Fig. 2d, tracking the characteristic peak near 1340 cm^−1^, the Raman signal reaches its maximum at the critical coupling strength, at which the cavity-antenna coupling strength ${g}_{{ca}}$ and the dissipation of the WGM microprobe (including the cavity intrinsic loss ${\Gamma }_{c0}$ and the fiber-coupled external loss ${\kappa }_{{\rm{in}}}$) are balanced, leading an optimized intracavity power and consequently the strongest excitation of nanoplasmonics. The theoretical derivation is found in Supplementary Section 1. Note that the experimental Raman signals at larger linewidth broadening are stronger than the theoretical expectation (solid curve). This can be explained by the increased number of excited nanoantennas and the stronger WGM scattering field at a smaller microprobe-substrate distance.

The pump laser was locked at the resonant frequency of the hybrid mode (${\Delta }_{{\rm{in}}}$ = 0) to keep the maximum excitation efficiency when acquiring Raman signals. With detuning, the signal degraded proportionally to the intra-power of the hybrid mode (Fig. 2e). In addition, a stronger Raman signal can be expected by further increasing the ratio of the external coupling strength ${\kappa }_{{\rm{in}}}$ to the intrinsic loss of WGM ${\Gamma }_{c0}$. The detuning ${\Delta }_{{\rm{in}}}$ determines the maximal light input when the cavity-antenna coupling is balanced with the system loss, while the ratio ${\kappa }_{{\rm{in}}}/{\Gamma }_{c0}$ reflects the capacity of accumulating light by the WGM microprobe. The signal maximums with different external coupling strengths were recorded and shown in Fig. 2f. More details about the optimization of Raman enhancement and the principle of selecting WGMs are found in Supplementary Sections 1 and 4.

**Fig. 3:**
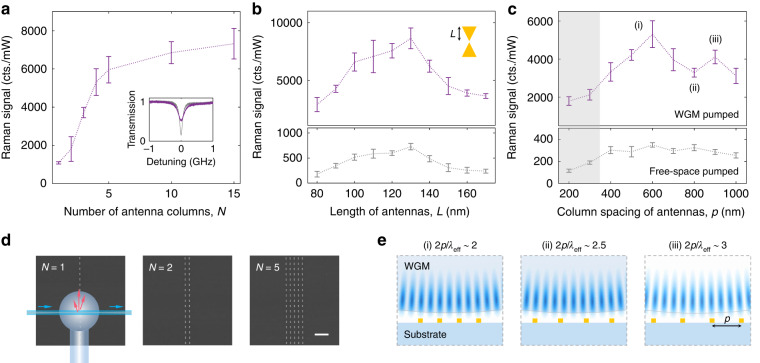
Engineering the coupling between WGM and nanoplasmonics. **a** Increased Raman signals with more nanoantennas coupled with the WGM microprobe. Inset: the WGM experiences a linewidth broadening when coupled with nanoplasmonics. **b**, **c** Raman signals enhanced by the nanoantennas with different (**b**) length ${L}$ and (**c**) column spacing ${p}$. The nanoplasmonic hotspots are pumped through (top panels) a WGM microprobe, i.e., forming the optoplasmonic hybrid mode, and (bottom panels) free-space light, respectively. The phase-matched cavity-antenna coupling ((i) and (iii)) leads to stronger light-matter interaction and Raman signals. **d** SEM images of nanoantenna columns and the top-view schematic of cavity-antenna coupling. For efficient excitation of hotspots, the axis of nanoantennas is perpendicular to the propagation direction and parallel to the electric field of WGMs. Scale bar: 2 μm. **e** Side-view schematic of phase-matched cavity-antenna coupling. At (i) and (iii), each nanoantenna is located at the maximum of the WGM electric field, and thus the Raman signals are maximized. The gold antennas are enlarged for illustration

Besides, the design of nanoantennas also influences the microprobe performance. As the number of nanoantennas coupled with the microsphere increases, the Raman signal initially grew and then saturated due to the limited effective WGM area on the tangential plane of the spherical cavity (Fig. 3a, d). The Raman enhancement from nanoplasmonics could be adjusted by the antenna geometry, such as length, width, and the separation between two triangles. Here, the effect of different lengths $L$ was demonstrated (Fig. 3b). Around 130 nm, the plasmonic resonance matches with the WGM ($\Delta$ = 0) as well as the input light wavelength, and thus the excitation efficiency to the target molecules is maximal. The measurements of extinction (Supplementary Fig. S6) confirmed the resonance matching.

### Phase-matched cavity-antenna coupling

Instead of random interactions of nanoantennas with the optical cavity, the microprobe configuration provides a platform to control the coupling between the WGM and nanoplasmonics. We propose a mechanism of phase-matched cavity-antenna coupling – each antenna precisely interacts with the WGM maximums – to further enhance the strength of the hybrid optoplasmonic modal field and thus the WGM sensor sensitivity. As known, nanoparticles (nanoantennas in this work) could introduce the coupling between clockwise- and counterclockwise-propagating WGMs, leading to a standing-wave field in the cavity^7^. The spacing of field maximums is theoretically ${\lambda }_{{\rm{eff}}}/2$, where ${\lambda }_{{\rm{eff}}}$ is the effective wavelength of the WGM. We define the case that each nanoantenna is located at the field maximums, i.e., the column spacing $p$ is an integer multiple of ${\lambda }_{{\rm{eff}}}/2$, as the phase-matched coupling condition (Fig. 3e). This condition maximizes the cavity-antenna coupling strength and thus the excitation efficiency of target Raman scattering (details in Supplementary Section 5).

In experiments, we find that there were two evident increases of Raman signals at $p$ = 600 nm (i) and 900 nm (iii) in Fig. 3c, while the corresponding free-space-pumped signals remained nearly unchanged. In contrast, a weaker Raman signal was observed with the mismatched coupling phase, *e.g*., $p$ = 800 nm (ii), showing that the phase-matched cavity-antenna coupling is advantageous to the performance of the WGM microprobe. Note that this phase-matching condition only contributes to the enhancement of the excitation field, while the spontaneous Raman scattering from molecules is still randomly phased. Additionally, in the shaded area of Fig. 3c, the deviation of plasmonic resonance occurred due to the interaction between adjacent antennas at smaller column spacing, which degrades the excitation efficiency and Raman signals. The proposed phase-matched cavity-antenna coupling approach could be leveraged to enhance coherent nonlinear processes (Supplementary Section 6).

### 2D Raman imaging

**Fig. 4:**
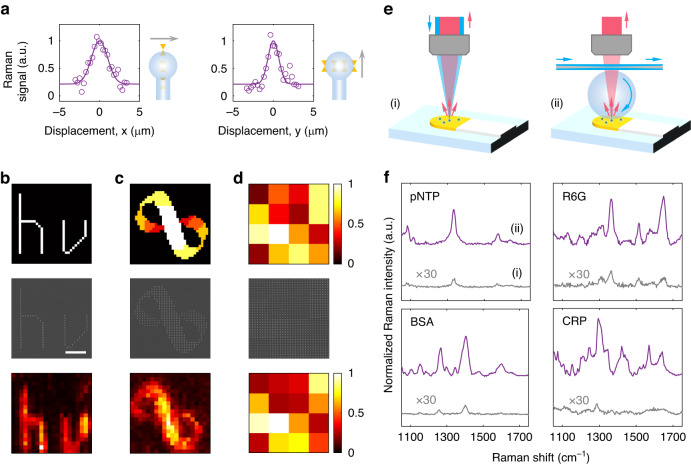
2D Raman imaging and enhancement of commercial SERS substrates through the WGM microprobe. **a** Normalized Raman signals as scanning over one-dimensional nanoantenna arrays in x- and y-direction, respectively. **b**–**d** 2D imaging of (**b**) Greek letters *hν* with uniform enhancement, (**c**) Mobius ring with varying enhancement, and (**d**) square grid with random enhancement. Top: normalized theoretical enhancements. Middle: SEM images. Bottom: normalized experimental signals. The variation of enhancements is realized by changing the length of nanoantennas ${L}$. The pixel sizes of **b**–**d** are 1 μm, 1 μm, and 5 μm, respectively. Scale bar: 5 μm. **e** Comparison between commercial SERS substrates (i) with traditional free-space optical pump and (ii) using the configuration of a WGM microprobe. The SERS substrates are the commercially available test papers with gold nanoparticles. **f** Enhanced Raman spectra of different molecules. pNTP 4-nitrothiophenol, R6G rhodamine 6G, BSA bovine serum albumin, CRP C-reactive protein

The scanning capability of the WGM microprobe extends the sensing area to an entire substrate. 2D mapping of enhanced Raman spectroscopy of sample substrates was demonstrated. The imaging resolution was evaluated by scanning a line of antenna arrays in x- and y-directions (Fig. 4a). The fitted linewidths, 2.38 μm and 1.57 μm, respectively, match the simulated mode size in the tangential plane of the microsphere. We designed three patterns for 2D Raman imaging: Greek letters *hν* with uniform enhancement, Mobius ring with varying enhancement, and square grid with random enhancement (Fig. 4b–d), where the enhancement strength was controlled by different antenna lengths $L$. The target molecules (pNTP) were bonded on the gold surface of each nanoantenna, corresponding to the bright pixels in the Raman images. The unbonded molecules were washed away by ethanol (Supplementary Section 3). The antenna spacing is set to 600 nm for phase-matched coupling. The Raman scattering of pNTP molecules was excited by the hybrid mode when the microprobe scanned a bright pixel. The 2D mapping of the NO_2_-group vibration indicated in the Raman spectra matches well with the designed enhancement distribution of target molecules. The WGM microprobe could also be used to measure an unknown enhancement distribution, like the randomly generated pattern (Fig. 4d).

### Improving commercial SERS substrates

Finally, we demonstrated the ability of the WGM microprobe to improve the sensitivity of commercial SERS test papers. As shown in Fig. 4e, the traditional use of commercial SERS test papers (i) was compared with the microprobe-based doubly enhanced Raman spectroscopy (ii). In addition to dye molecules, bovine serum albumin (BSA), a protein derived from cows, and C-reactive protein (CRP), an inflammatory marker travelling through the blood stream were also studied in this work (Fig. 4f). In experiments, the WGM microprobe improved the sensitivity of commercial SERS substrates by more than 100 times. The difference between the excitation areas in the two configurations was normalized. The sample preparation and the improvement of multiple SERS substrates can be found in “Methods” section and Supplementary Fig. S15.

## Discussion

The mechanisms for Raman enhancement include the pump enhancement, where Raman signals increase proportionally with the pump intensity, and the Purcell enhancement, which occurs at the Stokes emission wavelengths matching the resonances. In this microprobe configuration, the pump enhancement originates from both high-*Q* WGMs and nanoscale plasmonics, while the Purcell effect is mostly contributed by the broadband plasmonic resonance. Note that the Raman intensity would be further increased if the emission were also enhanced by WGMs. However, the narrow-linewidth WGMs modify the density of states and lead to the equidistant peaks at the emission band, which can be confused with the Raman peaks to be detected (Fig. S9). Therefore, the Purcell enhancement induced by WGMs should be avoided, and the obtained peaks in the spectra originate clearly from the Raman scattering of target molecules. The detailed investigation of different WGMs and different collection conditions can be found in Supplementary Section 4.

Generally, a stronger light-matter interaction can be acquired by (1) matched wavelength among pump light, WGM, and nanoplasmonic resonance (${\Delta }_{{\rm{in}}}$ = 0 and $\Delta$ = 0), (2) phase-matched cavity-antenna coupling, (3) appropriate polarization of the WGM, in which the electric field should be parallel to the axis of antennas, (4) fundamental WGM, of which the field evanescent overlapping with nanoantennas is maximum (Supplementary Section 4), (5) high-quality plasmonic substrate, for example, nanoantennas with a smaller mode volume ${V}_{a}$, (6) WGM with a lower intrinsic dissipation ${\Gamma }_{c0}$, and (7) optimal coupling distance $d$ and external coupling strength ${\kappa }_{{\rm{in}}}$. Based on the same experimental setup, the maximal Raman enhancement of 1.3 × 10^2^ can be achieved using the WGM microprobe if the ratio ${\Gamma }_{c0}/{\kappa }_{{\rm{in}}}$ further decreases close to zero (Supplementary Sections 1 and 4).

The highly controllable microprobe configuration enabled ultrahigh-sensitivity molecule detections with a phase-matched WGM-nanoplasmonic hybrid resonance, and allowed for a larger sensing area extended to the whole sample (comparison among existing sensors shown in Supplementary Fig. S16). The double signal enhancements and low optical power requirement hold promise for miniaturizing Raman spectroscopy and enabling broader applications with portable instruments. 2D Raman imaging was realized with a reusable microprobe thanks to little contamination and damage during non-contact scanning (spatial gap ~200 nm). Currently, the resolution of Raman imaging in this work is limited by the micron-level effective mode area of WGMs at the tangential plane with sample substrates. A deconvolution algorithm may provide an option for improving the resolution. We demonstrated the potential to realize the Raman imaging with subwavelength resolution by the WGM microprobe (Supplementary Fig. S13). For practical applications, the speed of Raman imaging can be improved by introducing a closed-loop operation^28^. The microprobe-substrate coupling distance can be automatically adjusted to optimize the signal collection efficiency and accelerate the imaging process by monitoring the transmission spectra of the hybrid resonance in real-time. In addition, due to the utilization of spontaneous Raman scattering, the imaging speed is restricted by the acquisition time ranging from milliseconds to several seconds per pixel. The enhancement of coherent stimulated Raman scattering by the WGM microprobe configuration is theoretically analyzed (Supplementary Section 6). The pixel dwell time could speed up to sub-milliseconds^29,30^.

The WGM microprobe configuration provides a practical and versatile solution to studying and exploring surfaces with interesting functions or properties. The enhanced light-matter interactions will facilitate nonlinear optics, including parametric oscillation, coherent spectroscopy, and comb generation. The flexibility will help the WGM microprobe become a novel tool for high-sensitivity detection and imaging, such as non-invasive analysis for 2D materials and enhanced photoluminescence of solid-state spin defects.

## Materials and methods

### Estimation of Raman enhancement factor

The normalized Raman enhancement factors (EFs) of nanoplasmonics (SERS) and WGM are given by

2 $$\overline{{{\rm{EF}}}_{{\rm{SERS}}}}=\left(\frac{{n}_{{\rm{SERS}},{\rm{FS}}}}{{P}_{{\rm{SERS}},{\rm{FS}}}\cdot {N}_{{\rm{SERS}},{\rm{FS}}}}\right)\Big/\left(\frac{{n}_{{\rm{bulk}}}}{{P}_{{\rm{bulk}}}\cdot {N}_{{\rm{bulk}}}}\right)$$

3 $$\overline{{{\rm{EF}}}_{{\rm{WGM}}}}=\left(\frac{{n}_{{\rm{SERS}},{\rm{WGM}}}}{{P}_{{\rm{SERS}},{\rm{WGM}}}\cdot {N}_{{\rm{SERS}},{\rm{WGM}}}}\right)\Big/\left(\frac{{n}_{{\rm{SERS}},{\rm{FS}}}}{{P}_{{\rm{SERS}},{\rm{FS}}}\cdot {N}_{{\rm{SERS}},{\rm{FS}}}}\right)$$

in which ${n}_{i}$ is the Stokes Raman photon counts integrated for 10 s, ${P}_{i}$ is the input power, and ${N}_{i}$ is the number of excited molecules. The intensity of Raman signals ${I}_{i}$ is defined as the ratio of ${n}_{i}/{P}_{i}$ (unit: cts./mW) in this paper. The subscriptions, “bulk,” “SERS, FS,” and “SERS, WGM,” correspond to the cases (i), (ii), and (iii) in Fig. 1c. The pump powers are 3 mW (10× integration time), 0.15 mW, and 0.15 mW, respectively. The insertion losses from the tapered fiber and beam splitter are eliminated when we characterize the pump power. The bulk sample of pNTP molecules used for the excitation without enhancement (Fig. 1c (i)) is prepared by evaporating its ethanolic solution (0.1 mM, 25 μL) on a glass slide and measured at the edge of the formed “coffee ring^31^”. The number of excited molecules (${N}_{{\rm{bulk}}}$ = 2.4 × 10^11^) is estimated from the ratio of the Gaussian beam spot size used for free-space pumping (19.7 μm^2^) to the “coffee-ring” area (0.126 mm^2^). On the other hand, the monolayer of pNTP molecules on the surface of gold nanoantennas ((ii) and (iii)) are formed with the help of Au-S chemical bonds (Supplementary Section 3). To maximize the EFs, an array of bowtie-shaped nanoantennas is fabricated with a column spacing $p$ = 600 nm and a row spacing $q$ = 780 nm. Each triangle is designed to be 120 nm long and 150 nm wide for plasmonic resonance. The number of excited molecules (${N}_{{\rm{SERS}},{\rm{FS}}}$ = 3.4 × 10^6^, ${N}_{{\rm{SERS}},{\rm{WGM}}}$ = 5.1 × 10^5^) is estimated based on the WGM excitation area (2.93 μm^2^, given by the linear scanning in Fig. 4) and the packing density of pNTP (0.22 nm^2^/molecule). Thus,

$$\overline{{{\rm{EF}}}_{{\rm{SERS}}}}=1.3\times {10}^{6},\,\overline{{{\rm{EF}}}_{{\rm{WGM}}}}=1.1\times {10}^{2}$$

and the total EF of the hybrid mode in the doubly enhanced Raman spectroscopy can be up to 10^8^ ($\overline{{{\rm{EF}}}_{{\rm{SERS}}}}\times \overline{{{\rm{EF}}}_{{\rm{WGM}}}}$). The Raman signals of SERS are increased about 16 times by WGM pumping without normalizing the numbers of excited molecules. The SNRs for (ii) and (iii) are calculated as 5.6 and 22.9, respectively, indicating that the SNR of free-space pumped SERS can be improved by about 4 times.

### Sample preparation for commercial SERS substrates

The SERS test papers deposited with gold nanoparticles were purchased from StellarNet, Inc. The pNTP and rhodamine 6G (R6G) solutions were prepared in ethanol and diluted to 0.01 mM and 0.1 mM, respectively. Bovine serum albumin (BSA) was dissolved in water at a concentration of 1 mM. The C-reactive protein (CRP) sample (0.37 mM) was purchased from Sigma-Aldrich. The analytes were stored in a refrigerator until experiments. 2.5 μL of each pNTP, R6G, BSA, and CRP solution were dropped on the SERS test papers and dried naturally. The coupling between the cavity and the SERS substrate was carefully adjusted to ensure strong Raman excitation, at which the tapered-fiber transmission was not reduced, and the optoplasmonic hybrid mode had a decent Q factor (>10^5^). The conditions for exciting Raman signals in Fig. 4f by the focused free-space light and the WGM microprobe remain the same (pump power 0.5 mW, acquisition time 10 s, and the same collection location). The enhancement ratios without normalizing the excited molecular numbers are calculated as 19, 14, 21, and 26 for the four analytes. All the tests achieved the sensitivity improvement of SERS substrates over 100 times.

### Supplementary information

Supplementary Information for A whispering-gallery scanning microprobe for Raman spectroscopy and imaging

## Data Availability

All data that support the findings in this study are available from the corresponding author upon reasonable request.
