# Supplementary material for: A whispering-gallery scanning microprobe for Raman spectroscopy and imaging
Source: Light Sci Appl. 2023 Oct 5;12:247. doi: 10.1038/s41377-023-01276-2 (PMC10556008; doi:10.1038/s41377-023-01276-2)
Supplement: Supplementary file 1 — Supplementary Information for A whispering-gallery scanning microprobe for Raman spectroscopy and imaging [file 41377_2023_1276_MOESM1_ESM.docx]

**Supplementary Information for**

**A whispering-gallery scanning microprobe for Raman spectroscopy and imaging**

**Wenbo Mao^1,†^, Yihang Li^1,†^, Xuefeng Jiang^1^, Zhiwen Liu^2^, Lan Yang^1,^***

1. *Department of Electrical and Systems Engineering, Washington University, St Louis, MO 63130, USA.*
2. *Department of Electrical Engineering, Pennsylvania State University, University Park, PA 16802, USA.*

† These authors contributed equally.

* Correspondence to: yang@seas.wustl.edu

**1. Theory of cavity-antenna coupling and field enhancement**

In this section, we derive the change of spectral features by using the cavity-antenna coupling theory in a linear-optic regime, such as frequency shift and linewidth broadening. Also, we discuss how the field enhancement of the WGM-nanoplasmonic hybrid mode can benefit the intensity of Raman scattering.


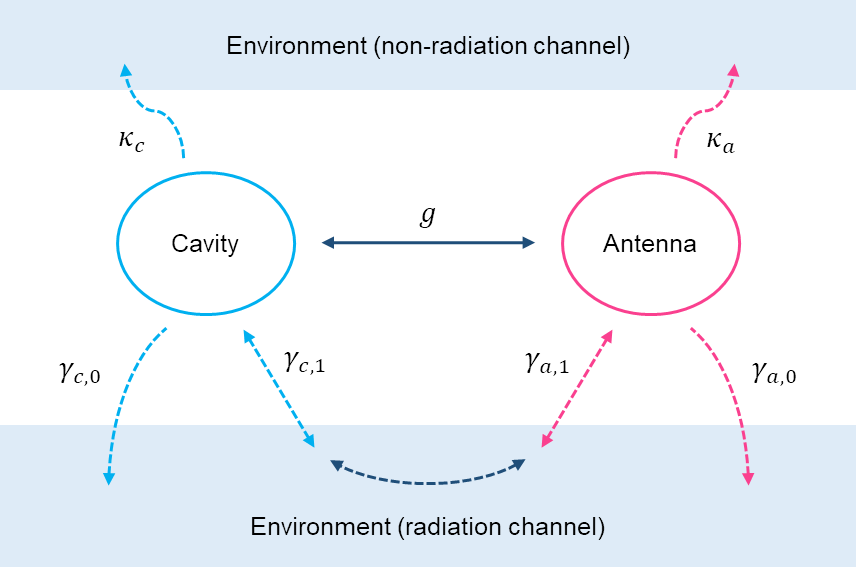


**Fig. S1 | Diagram of energy flow among cavity, antenna, and the environment.** The coupling between the cavity and antenna modes includes a field overlapping channel $g$ and a radiation channel $\sqrt{\gamma_{c,1}\gamma_{a,1}}$.

The Hamiltonian of a cavity-antenna coupled system can be written as

$$\begin{aligned} H=\Omega_{c}c^{\dagger}c+\Omega_{a}a^{\dagger}a+g\left( c^{\dagger}a+ca^{\dagger} \right)+\left( Gc^{\dagger}a-G^{*}ca^{\dagger} \right)\#\left( S1 \right) \end{aligned}$$

where $c$ and $a$ are the cavity mode (WGM) and antenna mode (nanoplasmonics), and $g$ ($G$) is the Hermitian (non-Hermitian) coupling strength between the two modes, respectively. The complex resonant frequencies are expressed by $\Omega_{c}=\omega_{c}-i\Gamma_{c}$ and $\Omega_{a}=\omega_{a}-i\Gamma_{a}$ as considering the mode dissipations. As shown in Fig. S1, the dissipations include non-radiation channels $\kappa_{c}$, $\kappa_{a}$ (*e.g.*, ohmic loss, absorption loss) and radiation channels $\gamma_{c,0}$, $\gamma_{a,0}$, $\gamma_{c,1}$, $\gamma_{a,1}$ ($\Gamma_{c}=\gamma_{c,0}+\gamma_{c,1}+\kappa_{c}$ and $\Gamma_{a}=\gamma_{a,0}+\gamma_{a,1}+\kappa_{a}$). The coupling channel $g$ results from the field overlapping under a dipole approximation,

$$\begin{aligned} g\approx-\frac{1}{2}f_{c}V_{0}\sqrt{\frac{\omega_{c}\omega_{a}}{\epsilon_{c}\epsilon_{a}V_{c}V_{a}}}\propto f_{c}\propto e^{-k^{'}\Delta d}\#\left( S2 \right) \end{aligned}$$

$f_{c}$ is the normalized field distribution of WGMs at the antenna location, $V_{0}$ is the spatial volume of antennas, and $V_{c}$ ($V_{a}$) is the mode volume of the cavity (antenna) mode. The interaction between the cavity and antennas is found exponentially decaying with the distance $\Delta d$ increasing (Fig. 2c) since $g$ is proportional to the WGM evanescent field $f_{c}$ at the location of antennas. The decay rate $k^{'}$ is a coefficient related to the cavity radius, refractive index, and mode order. Further discussion about phase-matched cavity-antenna coupling is provided in Supplementary Section 5. On the other hand, the non-Hermitian coupling ($G=i\sqrt{\gamma_{c,1}\gamma_{a,1}}e^{i\varphi}$) is given by the radiation pattern overlapping^1^. Generally, due to the localization of plasmonic modes, the coupling phase $\varphi$ is almost zero. We write down the rate equations of the coupled system according to the Heisenberg equation,

$$\begin{aligned} \left\{ \begin{aligned} i\frac{d}{dt}c=-i\Gamma_{c}c+\left( g+i\sqrt{\gamma_{c,1}\gamma_{a,1}}e^{i\varphi} \right)a \\ i\frac{d}{dt}a=\left( -\Delta-i\Gamma_{a} \right)a+\left( g+i\sqrt{\gamma_{c,1}\gamma_{a,1}}e^{-i\varphi} \right)c \end{aligned} \right.\#\left( S3 \right) \end{aligned}$$

in which the detuning between the WGM and plasmonic mode is defined as $\Delta=\omega_{c}-\omega_{a}$. To solve the eigenvalues of the coupled system, a stationary approximation is applied to the plasmonic mode due to their distinct characteristic timescales $\Gamma_{a}\gg\Gamma_{c}$. The rate equations are simplified as

$$\begin{aligned} i\frac{d}{dt}c=\left[ -i\Gamma_{c}+\frac{\left( g+i\sqrt{\gamma_{c,1}\gamma_{a,1}}e^{i\varphi} \right)\left( g+i\sqrt{\gamma_{c,1}\gamma_{a,1}}e^{-i\varphi} \right)}{\Delta+i\Gamma_{a}} \right]c\#\left( S4 \right) \end{aligned}$$

and then, the frequency shift $\delta\omega_{c}$ and linewidth broadening $\delta\Gamma_{c}$ of the cavity mode as coupled to plasmonics is given by

$$\begin{aligned} \delta\omega_{c}-i\delta\Gamma_{c}=\frac{g^{2}-\gamma_{c,1}\gamma_{a,1}+2ig\sqrt{\gamma_{c,1}\gamma_{a,1}}\cos\varphi}{\Delta+i\Gamma_{a}}\#\left( S5 \right) \end{aligned}$$

The coupling strengths can be solved from the experimentally measurable quantities,

$$\begin{aligned} g^{2}=\frac{1}{2}\sqrt{\left( \delta\omega_{c}^{2}+\delta\Gamma_{c}^{2} \right)\left( \Delta^{2}+\Gamma_{a}^{2} \right)}+\frac{\Gamma_{a}\delta\Gamma_{c}+\delta\omega_{c}\Delta}{2}\#\left( S6 \right) \end{aligned}$$

$$\begin{aligned} \gamma_{c,1}\gamma_{a,1}=\frac{1}{2}\sqrt{\left( \delta\omega_{c}^{2}+\delta\Gamma_{c}^{2} \right)\left( \Delta^{2}+\Gamma_{a}^{2} \right)}-\frac{\Gamma_{a}\delta\Gamma_{c}+\delta\omega_{c}\Delta}{2}\#\left( S7 \right) \end{aligned}$$

Now, we derive the field enhancement from the rate equations considering an external input $\epsilon_{p}=\sqrt{{2\kappa_{\mathrm{in}}P_{\mathrm{in}}}/{\hbar\omega_{p}}}$ with the coupling strength $\kappa_{\mathrm{in}}$, input power $P_{\mathrm{in}}$, and light frequency $\omega_{p}$,

$$\begin{aligned} \left\{ \begin{aligned} i\frac{d}{dt}c=\left( -\Delta_{p,c}-i\Gamma_{c}-i\kappa_{\mathrm{in}} \right)c+\left( g+i\sqrt{\gamma_{c,1}\gamma_{a,1}}e^{i\varphi} \right)a+i\epsilon_{p} \\ i\frac{d}{dt}a=\left( -\Delta_{p,a}-i\Gamma_{a} \right)a+\left( g+i\sqrt{\gamma_{c,1}\gamma_{a,1}}e^{-i\varphi} \right)c \end{aligned} \right.\#\left( S8 \right) \end{aligned}$$

where the mode detuning is defined as $\Delta_{p,c}=\omega_{p}-\omega_{c}$ and $\Delta_{p,a}=\omega_{p}-\omega_{a}\approx\Delta$. Similarly, the stationary approximation gives the field intensities,

$$\begin{aligned} \left| c \right|^{2}=\frac{\epsilon_{p}^{2}}{\left( \Delta_{p,c}-\delta\omega_{c} \right)^{2}+\left( \Gamma_{c}+\delta\Gamma_{c}+\kappa_{\mathrm{in}} \right)^{2}}\#\left( S9.1 \right) \end{aligned}$$

$$\begin{aligned} \left| a \right|^{2}=\frac{g^{2}+\gamma_{c,1}\gamma_{a,1}}{\Delta^{2}+\Gamma_{a}^{2}}\frac{\epsilon_{p}^{2}}{\left( \Delta_{p,c}-\delta\omega_{c} \right)^{2}+\left( \Gamma_{c}+\delta\Gamma_{c}+\kappa_{\mathrm{in}} \right)^{2}}\#\left( S9.2 \right) \end{aligned}$$

which can be converted to electric fields by

$$\begin{aligned} \left| \boldsymbol{E}_{\boldsymbol{c}} \right|^{2}=\frac{\hbar\omega_{p}\left| c \right|^{2}}{\epsilon_{0}\epsilon_{c}V_{c}}\#\left( S10.1 \right) \end{aligned}$$

$$\begin{aligned} \left| \boldsymbol{E}_{\boldsymbol{a}} \right|^{2}=\frac{\hbar\omega_{p}\left| a \right|^{2}}{\epsilon_{0}V_{a}}\#\left( S10.2 \right) \end{aligned}$$

In the experiments, we compare the performance of the optoplasmonic (WGM-nanoplasmonics) hybrid modes with that of free-space-pumped nanoplasmonic modes. We introduce a free-space coupling strength $\kappa_{F}$, indicating the efficiency of the free-space light exciting the nanoplasmonic modes. The rate equation is rewritten as

$$\begin{aligned} i\frac{d}{dt}a^{\left( F \right)}=\left( -\Delta-i\Gamma_{a} \right)a^{\left( F \right)}+i\sqrt{\frac{2\kappa_{F}P_{\mathrm{in}}}{\hbar\omega_{p}}}\#\left( S11 \right) \end{aligned}$$

and

$$\begin{aligned} \left| \boldsymbol{E}_{\boldsymbol{a}^{\left( \boldsymbol{F} \right)}} \right|^{2}=\frac{\hbar\omega_{p}\left| a^{\left( F \right)} \right|^{2}}{\epsilon_{0}V_{a}}=\frac{2\kappa_{F}}{\epsilon_{0}V_{a}}\frac{1}{\Delta^{2}+{\Gamma_{a}}^{2}}P_{\mathrm{in}}\#\left( S12 \right) \end{aligned}$$

It shows that the enhanced optical field for Raman excitation is linear to the pump power $P_{\mathrm{in}}$ (Fig. 1d, inset). By substituting the Eqs. (S6), (S7), (S9.2), (S10.2), and (S12), the enhancement factor (EF) of WGM pumping, a ratio of the WGM-pumped plasmonic intensity to the free-space-pumped plasmonic intensity, is obtained by

$$\begin{aligned} \eta=\frac{\left| \boldsymbol{E}_{\boldsymbol{a}} \right|^{2}}{\left| \boldsymbol{E}_{\boldsymbol{a}^{\left( \boldsymbol{F} \right)}} \right|^{2}}=\frac{\kappa_{\mathrm{in}}}{\kappa_{F}}\frac{\sqrt{\left( {\delta\omega}_{c}^{2}+{\delta\Gamma}_{c}^{2} \right)\left( \Delta^{2}+\Gamma_{a}^{2} \right)}}{\left( \Delta_{p,c}-\delta\omega_{c} \right)^{2}+{\Gamma_{c}^{'}}^{2}}\#\left( S13 \right) \end{aligned}$$

Where $\Gamma_{c}^{'}\equiv\Gamma_{c}+\delta\Gamma_{c}+\kappa_{\mathrm{in}}$ is the total loss of the hybrid mode, which can be measured by the linewidth from the transmission spectrum of the optoplasmonic hybrid mode. The enhancement factor $\eta$ shows a Lorentzian relation with regard to the detuning of the pump light, $\Delta_{p,c}-\delta\omega_{c}$, which was demonstrated in Fig. 2e of the main text. Note that $\Gamma_{c}$ includes both the cavity intrinsic loss $\Gamma_{c0}$ and the radiation loss due to leaking to the glass substrate, while $\delta\Gamma_{c}$ is the loss induced by the cavity-antenna coupling.

To derive the variation tendency of Raman signals, we assume $\delta\omega_{c}\approx\beta\delta\Gamma_{c}$ and separate $\Gamma_{c0}$ from $\Gamma_{c}$, *i.e.*, $\Gamma_{c}^{'}\approx\Gamma_{c0}+\alpha\delta\Gamma_{c}+\kappa_{\mathrm{in}}$, where $\alpha$, $\beta$ are constant coefficients, $\Gamma_{c0}$ is the intrinsic loss of the cavity without antennas coupled, and $\alpha\delta\Gamma_{c}$ represents the linewidth broadening including the effects from both antennas and the substrate. The assumption is based on the fact that $\delta\omega_{c}$, $\delta\Gamma_{c}$, and $\Gamma_{c}$ are all exponentially related to the cavity-substrate distance $\Delta d$ with the close spatial decay rates (Eq. (S2) and Fig. 2c). At the zero detuning $\Delta_{p,c}=\delta\omega_{c}$, we have

$$\begin{aligned} \eta\approx\frac{\kappa_{\mathrm{in}}}{\kappa_{F}}\frac{\sqrt{\left( 1+\beta^{2} \right)\left( \Delta^{2}+\Gamma_{a}^{2} \right)}}{\left( \frac{\Gamma_{c0}+\kappa_{\mathrm{in}}}{\sqrt{\delta\Gamma_{c}}}+\alpha\sqrt{\delta\Gamma_{c}} \right)^{2}}\#\left( S14 \right) \end{aligned}$$

which implies the existence of a maximum $\eta_{\max}$ with regard to the linewidth broadening of the hybrid mode $\delta\Gamma_{c}$ when the nanoantennas approach the cavity,

$$\begin{aligned} \eta_{\max}\approx\frac{\sqrt{\left( 1+\beta^{2} \right)\left( \Delta^{2}+\Gamma_{a}^{2} \right)}}{4\alpha\kappa_{F}}\frac{\kappa_{\mathrm{in}}}{\Gamma_{c0}+\kappa_{\mathrm{in}}}\#\left( S15 \right) \end{aligned}$$

The results were experimentally demonstrated in Fig. 2d. Furthermore, the $\eta_{\max}$ can be improved by reducing the intrinsic loss $\Gamma_{c0}$ or increasing the external coupling strength $\kappa_{\mathrm{in}}$, as described in Fig. 2f and the discussion section of the main text. The condition of ${\kappa_{\mathrm{in}}}/{\Gamma_{c0}}\gg1$ can push the EF to a theoretical maximum,

$$\begin{aligned} \eta_{\max}\to\frac{\sqrt{\left( 1+\beta^{2} \right)\left( \Delta^{2}+\Gamma_{a}^{2} \right)}}{4\alpha\kappa_{F}}\#\left( S16 \right) \end{aligned}$$

In Fig. S2, we plot the changed Raman signals by adjusting the distance $\Delta d$ between the cavity and antennas at three different external coupling strengths $\kappa_{\mathrm{in}}$. A larger Raman signal could be expected by stronger external coupling in principle.


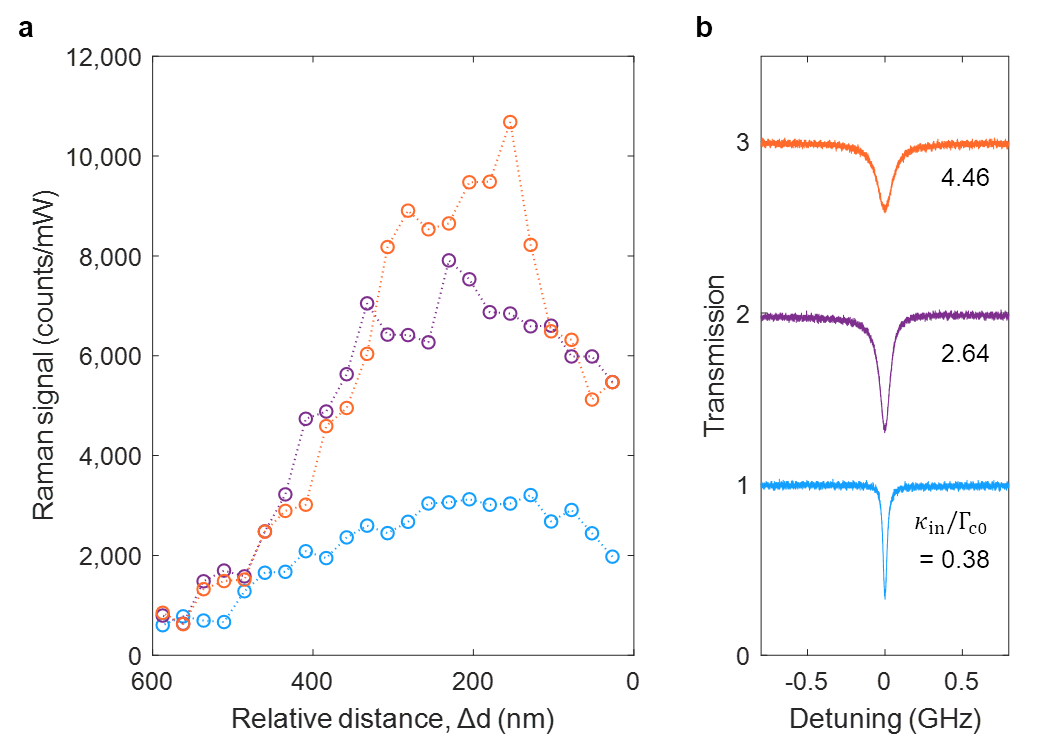


**Fig. S2 | Raman signals at different external coupling strengths.** **a**, Raman signals as the distance between the microsphere cavity and nanoantennas decreases. The colors correspond to three different external coupling strengths $\kappa_{\mathrm{in}}$ in **b**. **b**, Transmission spectra of WGMs before the nanoantennas approach. $\Gamma_{c0}$, intrinsic loss of the WGM.

**2. Experimental setup for doubly enhanced Raman spectroscopy**

We design and build an experimental setup (Fig. S3). The target molecules can be optically pumped from either the WGM microprobe through a tapered fiber waveguide (A1) or the free-space focused light (A2). The free-space optical path (B) is used to collect the Raman scattering.

To achieve the WGM-nanoplasmonic hybrid resonance for doubly enhanced Raman spectroscopy, a sandwich configuration arranges the tapered fiber, silica microsphere cavity, and substrate with nanoantennas from top to bottom. The cavity and substrate are mounted on two piezo-driven nano-stages separately. To excite the hybrid resonance, the input light (780-nm band) from a fiber-coupled external-cavity tunable laser (Newport TLB-6712), after adjusting the light intensity and polarization, is coupled into the WGM microprobe via a tapered fiber (A1). The nanoplasmonic hotspots are excited by the evanescent field of the WGM, enhancing the Raman scattering of target molecules. The transmission spectra of the hybrid resonances are monitored by the photodetector (PD1). As a comparison, the other free-space pump path (A2) is available in this setup. The input light from the same tunable laser is collimated and focused onto the sample through a long-working-distance objective (NA = 0.55).

The spontaneous Raman scattering is collected by the same objective in the free-space path (B). The Stokes light is separated by an 805-nm dichroic mirror. An 800-nm long-pass filter is used to further remove the residual pump light, after which the Raman spectra are recorded by a liquid-nitrogen-cooled spectrometer (Princeton Instrument Acton SP2500). In addition, the polarization of the pump light is examined by PD2 in a side path.

We show the microscope images of the sandwich configuration in Fig. S4. The positions of the cavity and substrate can be separately adjusted to optimize the coupling conditions. In the experiments, the tapered fiber was always in contact with the microsphere for mechanical stability, and the external coupling strength $\kappa_{\mathrm{in}}$ was tuned by carefully selecting the fiber diameter at the coupling point.


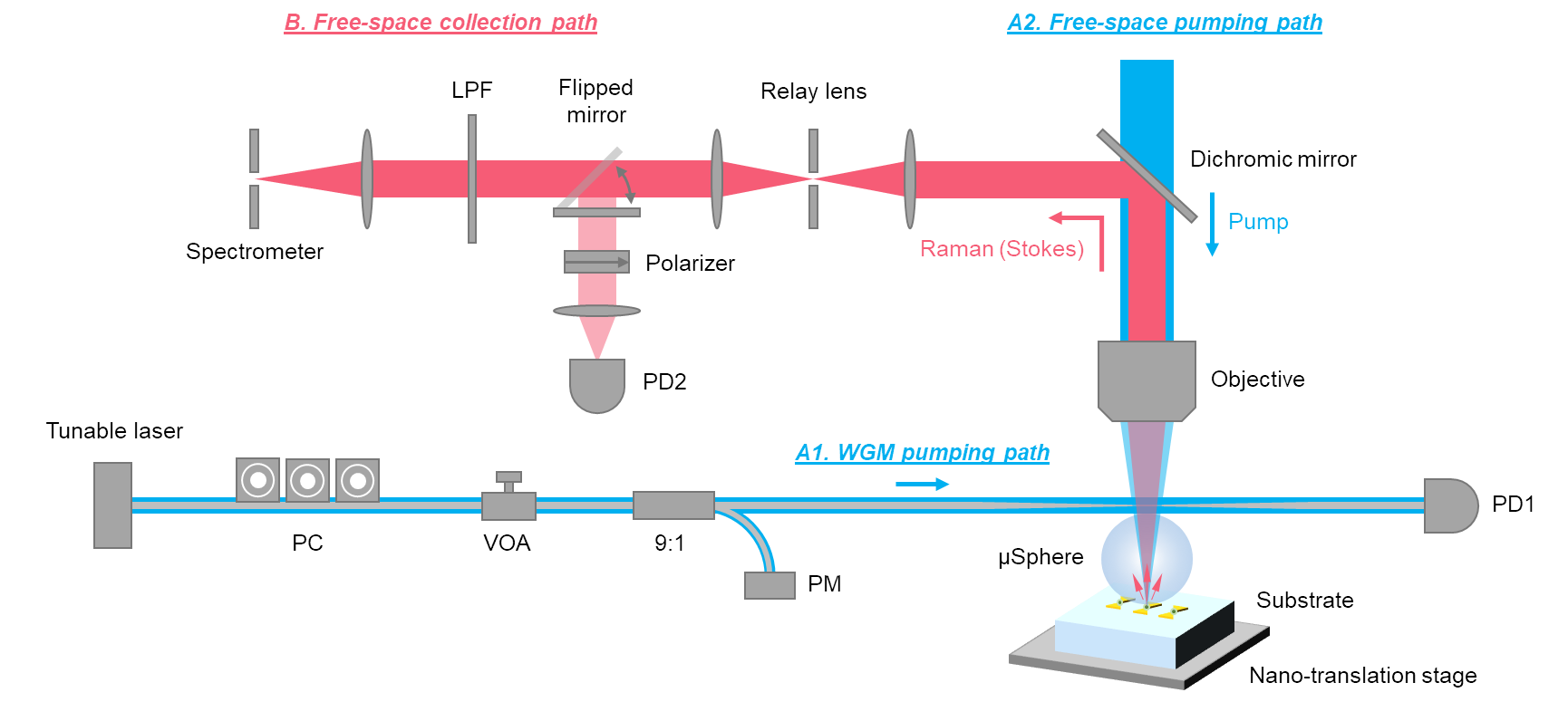


**Fig. S3 | Schematic of the experimental setup.** Target molecules on the substrate can be pumped by a tapered-fiber-coupled WGM (A1) or by focused free-space light (A2). The Raman scattering is collected by free-space path (B). PC, polarization controller; VOA, variable optical attenuator; PM, power meter; μSphere, microsphere; PD, photodetector; LPF, long-pass filter.


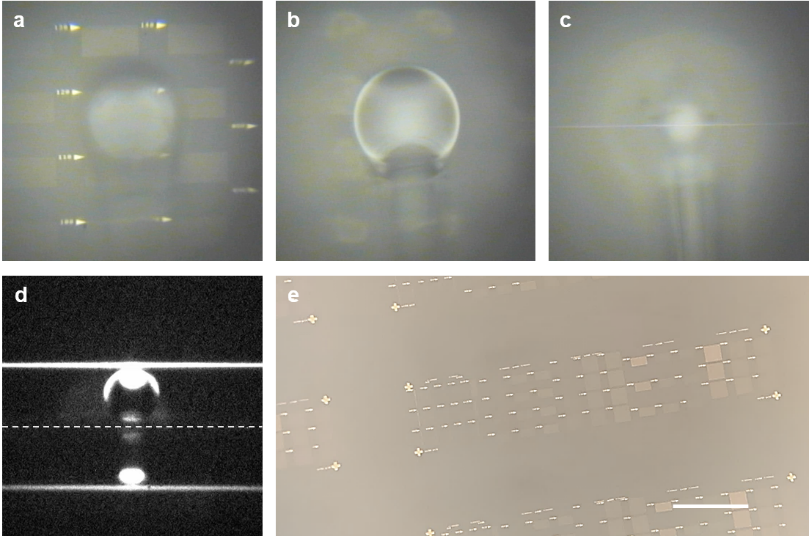


**Fig. S4 | Microscope images of the fiber-cavity-antenna sandwich setup.** **a**, **b**, **c**, Top views showing the substrate, cavity, and tapered fiber, respectively. **d**, Side view. The dashed line marks the substrate interface. **e**, Multifunctional substrate with different nanoantennas. Scale bar: 100 μm.

**3. Fabrication of bowtie-shaped nanoantennas**

This section describes the procedures of fabricating nanoantennas on a glass substrate and depositing a monolayer of target molecules (4-nitrothiophenol, pNTP) (Fig. S5). Also, we characterize the extinction spectra of nanoantennas by a UV-vis spectrometer to determine the plasmonic resonance (Fig. S6).

**Step 1**: The fused silica wafer (10 mm × 5 mm) was ultrasonically cleaned with acetone and isopropyl alcohol (IPA), followed by deionized water rinsing and oxygen plasma cleaning. Hexamethyldisilazane (HMDS) was thermally evaporated as an adhesion layer between the glass and the resist. For clean sidewalls, the bilayer e-beam resists of methyl methacrylate (MMA, 100 nm) and polymethyl methacrylate (PMMA, 50 nm) were used to define the patterns. Each layer was baked for 3 minutes at 180 °C on a hot plate. A layer of gold (6 nm) was deposited for anti-charging before electron-beam lithography (EBL).

**Step 2**: The pattern of bowtie-shaped nanoantennas was defined by EBL (Elionix ELS-S50EX) with 50 kV and 100 pA. Before developing in methyl isobutyl ketone (MIBK, 1:3) at room temperature, the conductive gold layer was removed by potassium-iodide-based etchant. The resulting bilayer resist with a larger undercut is helpful for the following lift-off process.

**Step 3**: The 3-nm chromium and 30-nm gold were deposited by thermal evaporation with an initial pressure of 5×10^-7^ mBar and deposition rate of 0.7 Å s^-1^. The chromium layer improves the adhesion between gold and glass.

**Step 4**: The lift-off process was done by soaking the sample in acetone at room temperature for 2 hours, then ultrasonic cleaning twice for 60 seconds, and rinsing in IPA and deionized water. The desired patterns were left on the glass substrate.

**Step 5**: The monolayer of target molecules (pNTP) was deposited with the help of Au-S chemical bonds. A short oxygen plasma treatment (50 W, 2 minutes) was applied to remove contaminants and make the surface hydrophilic. The sample was immersed overnight in a pNTP/ethanol solution for labeling. Finally, we washed off the excessive (unbonded) molecules with ethanol and water.


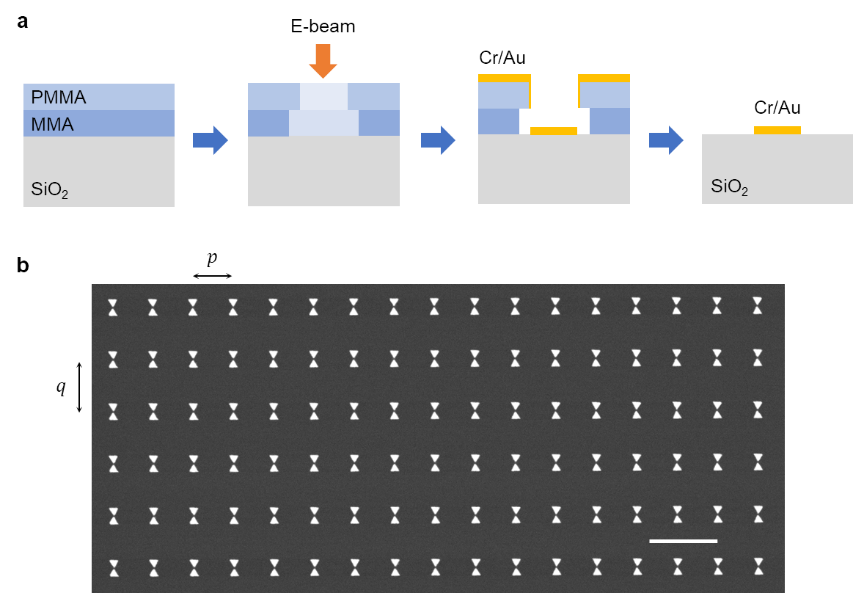


**Fig. S5 | Fabrication of bowtie-shaped nanoantennas.** **a**, Fabrication procedures. **b**, Fabricated nanoantennas with column spacing $p$ and row spacing $q$. Scale bar: 1 μm.


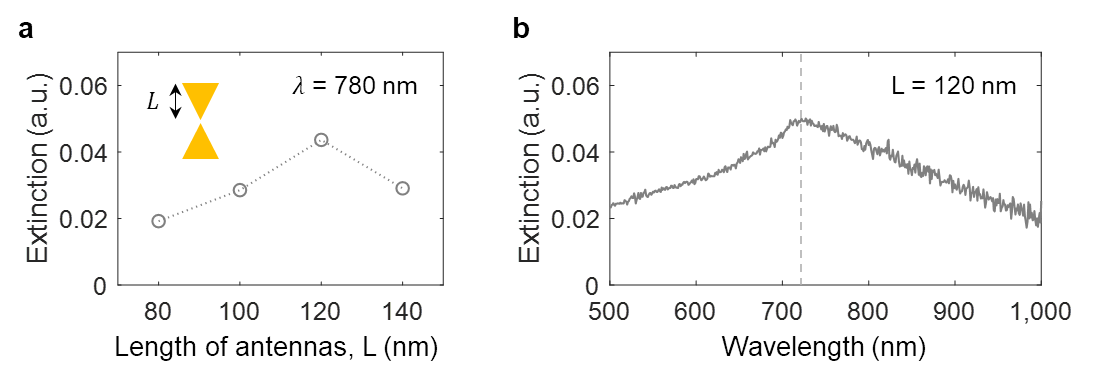


**Fig. S6 | Extinction spectra of nanoantennas measured by a UV-vis spectrometer.** **a**, Extinction (incident wavelength 780 nm) of nanoantennas with different lengths. **b**, Extinction spectra of nanoantennas with a length of 120 nm.

**4. Choice of WGMs for effectively coupling with antennas**

The cavity-antenna coupling, used to form the optoplasmonic hybrid mode, is critical to enhance the localized field in hotspots (Supplementary Section 1). More efficient field overlap results in stronger light-matter interactions and stronger Raman signals. The WGMs in a microsphere with different polarizations and field distributions (mode orders) are simulated by a 2D axisymmetric finite-element-method as shown in Fig. S7. The polarization is classified as transverse magnetic (TM) and transverse electric (TE), which is marked by the arrows. In our experiments, the electric field of TM-polarized modes is parallel to the long axis of antennas, while that of TE-polarized modes is perpendicular to the substrate plane. The spatial field distribution is described by radial mode number $n$ and polar mode number $l$, respectively.

The nanoplasmonic hotspots are only excited by the electric field parallel to the long axis of antennas (Fig. S8a). The Raman signal of target molecules excited by free-space light with perpendicular electric fields was hardly collected. Interestingly, a weak but measurable Raman signal was obtained with TE-polarized WGM pumping (Fig. S8b). We attribute this phenomenon to the presence of a parallel component due to light scattering induced by the antennas or the cavity surface non-uniformity.


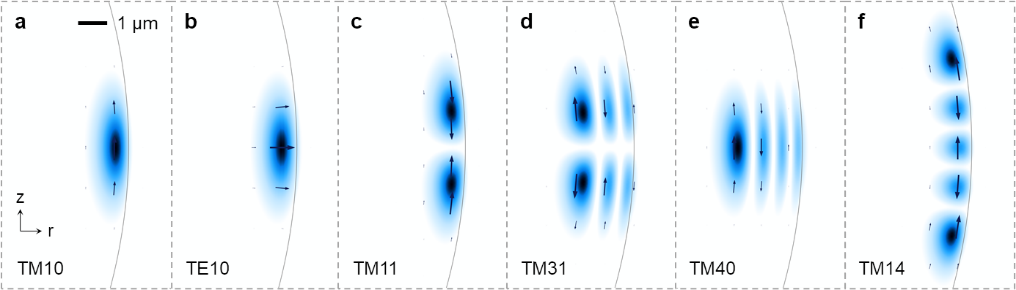


**Fig. S7 | 2D axisymmetric simulation of WGMs.** The figures show the norm of electric fields in a microsphere. The propagation direction of WGMs is perpendicular to the plane. Example: TM10, TM-polarized mode with $n$ = 1 and $l$ = 0.

There are two effects contributing to the enhancement of Raman signals^2–4^, including enhanced excitation of pump light and enhanced emissions due to modified density of states (Purcell effect). The excitation field in the microprobe configuration has double enhancements resulting from the high-Q WGMs and nanoscale plasmonics, respectively. However, the enhancement due to the Purcell effect mostly depends on the choice of WGMs. For example, in Fig. S9c, there is no narrow spike with WGMs spectral signatures observed, implying that the emission modification from WGMs is negligible due to the weak cavity-antenna coupling at the emission band. On the other hand, with different orders of WGMs chosen, in Fig. S9d, the equidistant spikes indicate that the density of states is modified by WGMs at the emission band, *i.e.*, the Purcell effect. The periodicity agrees with the free spectral range (FSR) of the microsphere used in this work.

Note that the observation of these spikes is also relevant to the collection location (Figs. S9a and S9b). The spikes are much more prominent when signals are collected from the edge of the microsphere than from the center, because the WGMs propagate in the same direction as the collection direction at the edge^2^.


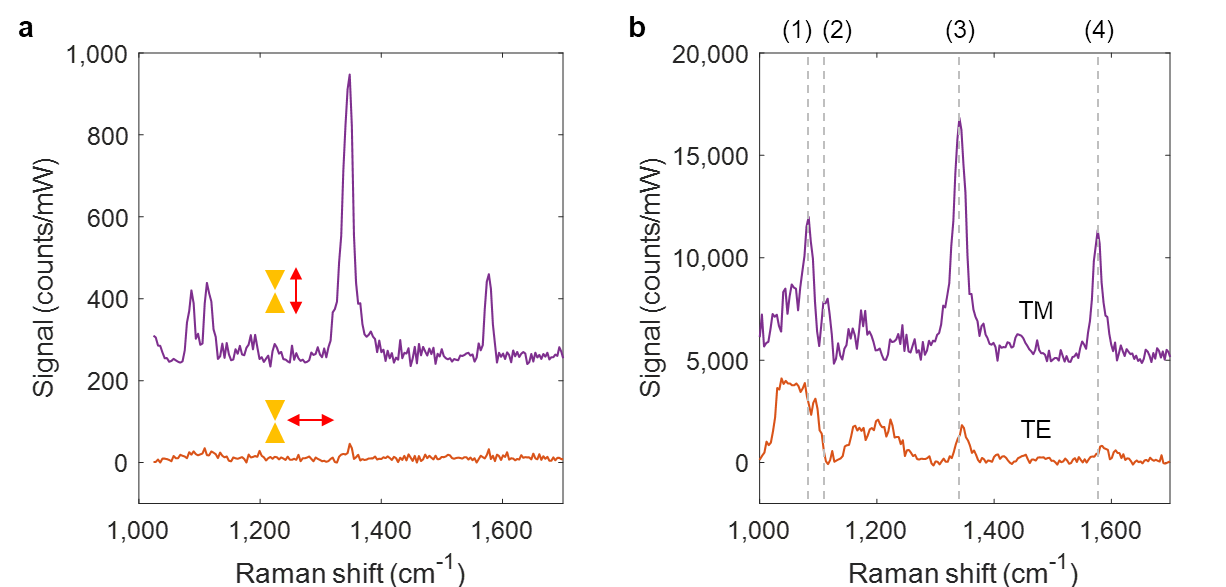


**Fig. S8 | Polarization-dependent Raman spectra.** **a**, Raman spectra enhanced by free-space-pumped nanoplasmonics. **b**, Raman spectra enhanced by the WGM microprobe with transverse magnetic (TM) polarization and transverse electric (TE) polarization, respectively.


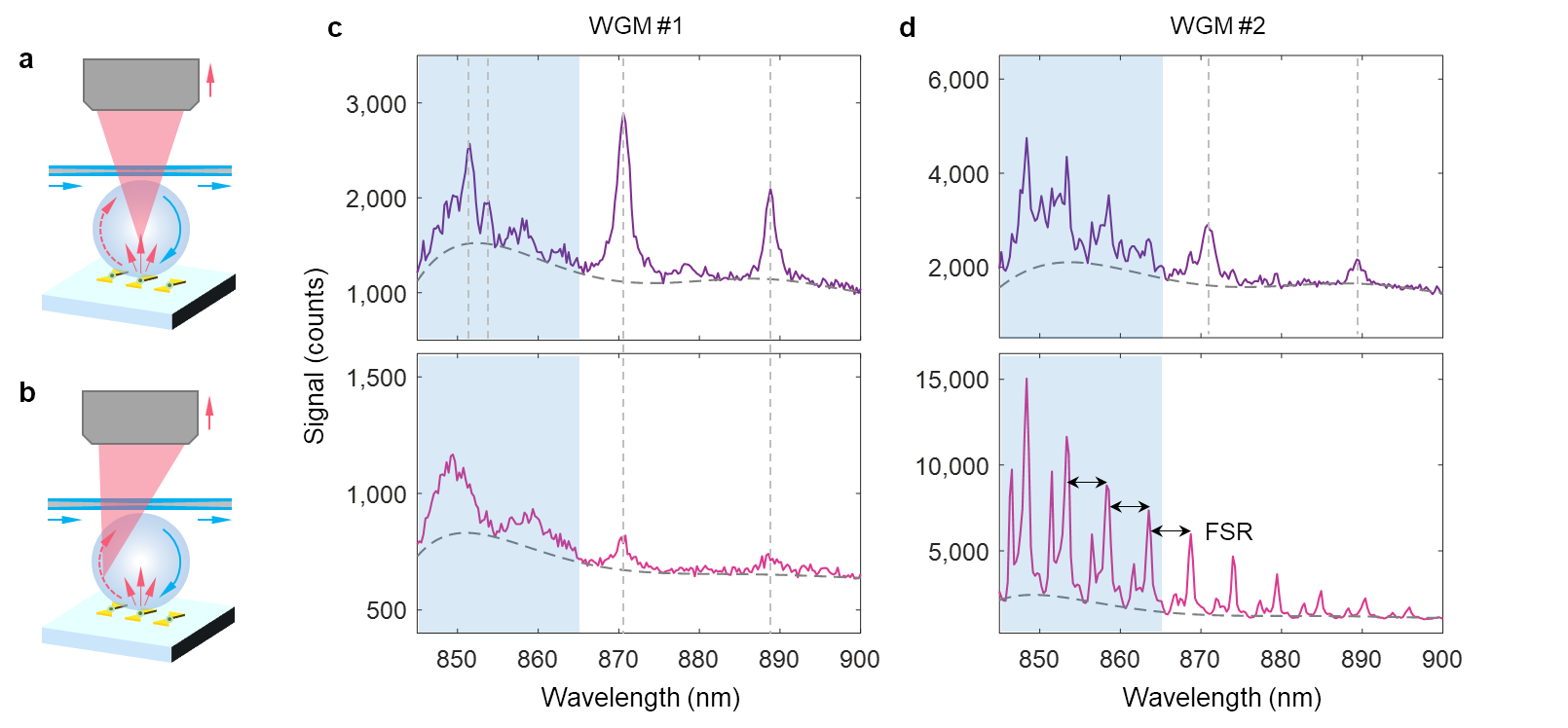


**Fig. S9 | Collection of Raman scattering from different locations.** **a**, **b**, Schematic of the collection from the (**a**) center and (**b**) edge of the microsphere. **c**, **d**, Emission spectra as pumped by (**c**) WGM #1 and (**d**) WGM #2 and collected from the center (top panels) and edge (bottom panels) of the microsphere, respectively. The free-space spectrometer is focused on the substrate plane for each case. FSR, free spectral range.

In addition, we find that the spectra are influenced by orders of WGMs, *i.e.*, field distribution. We consider the Raman signals pumped by two different WGMs and collected from the center and edge, respectively. For WGM #1, the edge-collected signal was weaker than the center-collected signal. The molecular Raman scattering coupled into the cavity was weak, since the spontaneous Raman scattering has random optical phases; otherwise, there would be equidistant WGM spikes at the emission band, indicating the Purcell effect with WGM-modified density of states.

On the other hand, the strong Purcell effect of enhanced Raman scattering was observed in the WGM #2. Note that the WGM-modified Raman emission was mainly from silica instead of target molecules (pNTP) in hotspots, which is implied from the fact that the spikes mainly appear around silica Raman shifts (shading areas). We believe, in this case, the WGM #2 is a high-order mode with the field maximum localized deeply inside the cavity. The excitation efficiency of the silica material of the cavity itself is much higher than that of the target molecules in hotspots. The Raman scattering from silica was strongly enhanced with WGMs by the Purcell effect and exhibited equidistant spikes.

**5. Phase-matched cavity-antenna interaction**

In this section, we derive the condition of forming standing-wave WGMs in a microcavity coupled with multiple nanoantennas and simulate the antenna field intensity of phase-matched (PM) and non-phase-matched (non-PM) coupling, such as the coupling with a single antenna and randomly distributed antennas.


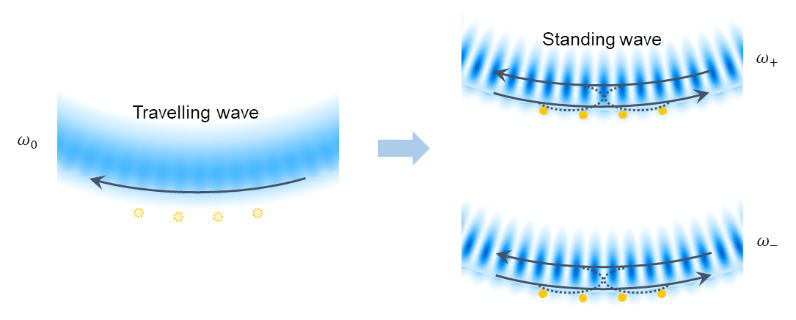


**Fig. S10 | Formation of standing waves in a microcavity.** The travelling wave ($\omega_{0}$) is supported in a microcavity without nanoantennas coupled. Two supermodes ($\omega_{\pm}$) are formed as the cavity is coupled with nanoantennas as a result of the interaction between CW- and CCW WGMs. The antennas are located at the antinodes and nodes of the standing waves, respectively. The nanoantennas are modeled by four gold nanospheres with a diameter of 10 nm, separated from the cavity boundary by 50 nm.

A microcavity intrinsically supports pairs of degenerate modes ($\omega_{0}$), *i.e.*, travelling-wave WGMs propagating in clockwise (CW) and counterclockwise (CCW) directions. The CW (CCW) mode can be selectively excited by coupling light to the resonator from different directions. As nanoparticles (nanoantennas) perturb the cavity, the coupling channel between the CW and CCW modes is established, and the standing-wave supermodes are formed in the cavity^5^ (Fig. S10). The cavity-antenna interaction maximizes when the antennas are located at the antinodes of the standing wave. For a cavity perturbed by $N$ nanoparticles, the CW-CCW interaction Hamiltonian can be written as

$$\begin{aligned} H_{I}=\left( \begin{matrix} & \tilde{\mu}\sum_{n=1}^{N} e^{iknp} \\ \tilde{\mu}\sum_{n=1}^{N} e^{-iknp} & \end{matrix} \right)\#\left( S17 \right) \end{aligned}$$

in which $\tilde{\mu}$ is the complex perturbation of each antenna, $k$ is the wavevector of WGMs ($k={2\pi n_{\mathrm{eff}}}/\lambda$), and $p$ is the spacing of antennas. The eigenmodes are the superposition of CW- and CCW-propagated lights with the eigenfrequencies

$$\begin{aligned} \omega_{\pm}=\pm\tilde{\mu}\left| \frac{\sin\frac{Nkp}{2}}{\sin\frac{kp}{2}} \right|\#\left( S18 \right) \end{aligned}$$

Intuitively, the standing wave can enhance the maximum field intensity by two times ($\left( \psi_{\mathrm{CW}}\pm\psi_{\mathrm{CCW}} \right)/\sqrt{2}$). However, the practical coupling strength and cavity dissipation significantly influence the proportion of standing-wave WGMs. For example, as shown in Fig. S11a, the phase-matching advantage, defined as the ratio of PM intensity to non-PM intensity, degrades with the increased cavity-antenna distance due to a weakened CW-CCW coupling and standing-wave proportion.

As the spacing of antennas changes (Fig. S11b), the field intensity reaches the maximums at the PM coupling ($p$ = 300, 600, 900 nm, with arrows). The cases in which the antennas are located at the node of standing-wave WGMs ($\omega_{-}$) show weaker field intensities. The antenna array is modeled by four nanospheres, and the cavity-antenna coupling distance is kept at 50 nm. The total energy is normalized in the simulations. Note that the field intensity increases with more antennas coupled (Fig. S11c). The previously reported WGM-nanoplasmonic hybrid sensing platforms^6–9^ were realized with non-PM interaction because the distribution of nanoantennas was random and hard to control. The enhanced cavity-antenna interaction by phase matching in this work can further benefit the molecular sensing performance. The improvement has been experimentally verified in Fig. 3c of the main text. The average Raman signal at (i) was roughly 1.6 times stronger than at (ii).


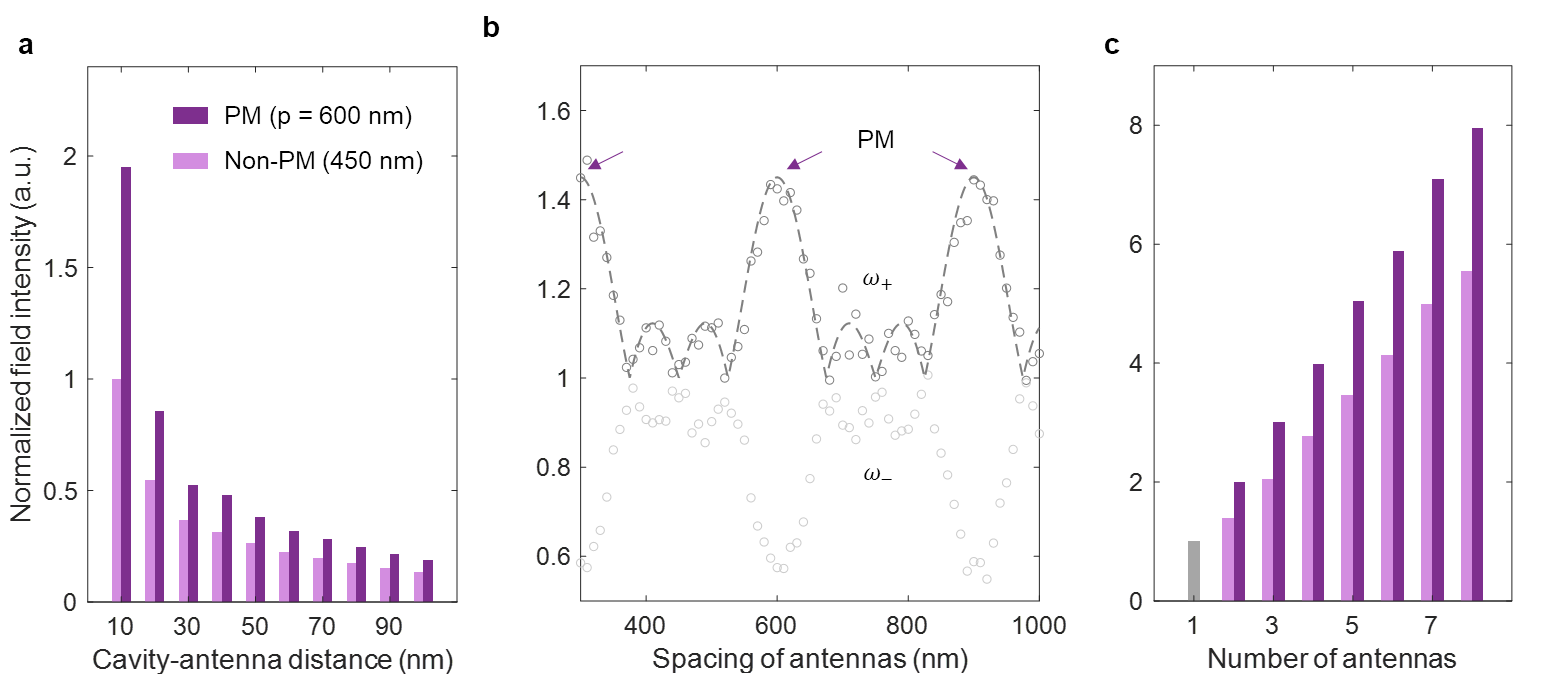


**Fig. S11 | Simulation of field intensity at the antenna location.** The cavity-antenna coupling is simulated with different (**a**) distances, (**b**) spacings of antennas, and (**c**) numbers of antennas. The phase-matched (PM) and non-phase-matched (non-PM) coupling are realized by changing the spacing, $p=\lambda_{\mathrm{eff}}$ and $p={3\lambda_{\mathrm{eff}}}/4$, respectively. The antenna arrays used in (**a**) and (**b**) are modeled by 4 nanospheres.

**6. WGM-microprobe-enhanced stimulated Raman spectroscopy**

This work has demonstrated Raman spectroscopy based on enhanced spontaneous Raman scattering by a WGM microprobe. Due to the random phase of the scattering light, the potential for the greatly enhanced light-matter interaction by the WGM microprobe has not been fully exploited. The enhancement provided by the WGM microprobe can be extended to various nonlinear optic processes. Here, we present a theoretical analysis of enhanced stimulated Raman spectroscopy (SRS) using the WGM-nanoplasmonics hybrid resonance.

The equations describing the cavity-antenna coupling follow Supplementary Section 1. For simplicity, the indirect coupling channel $\gamma_{c,1}\gamma_{a,1}$ is neglected and the Raman gain of target molecules is written as a constant $g_{R}$. We use the subscriptions *P* and *R* to represent the pump and Stokes lights, respectively.

$$\begin{aligned} \left\{ \begin{aligned} i\frac{d}{dt}c_{P}=\left( -\Delta_{P}-i\Gamma_{c,P}-i\kappa_{\mathrm{in},P} \right)c_{P}+ga_{P}+i\epsilon_{P} \\ i\frac{d}{dt}a_{P}=\left( -\Delta-i\Gamma_{a} \right)a_{P}+gc_{P}-i\frac{\omega_{P}}{\omega_{R}}g_{R}\left| a_{R} \right|^{2}a_{P} \\ i\frac{d}{dt}c_{R}=\left( -\Delta_{R}-i\Gamma_{c,R}-i\kappa_{\mathrm{in},R} \right)c_{R}+ga_{R}+i\epsilon_{R} \\ i\frac{d}{dt}a_{R}=\left( -\Delta-i\Gamma_{a} \right)a_{R}+gc_{R}+ig_{R}\left| a_{P} \right|^{2}a_{R} \end{aligned} \right.\#\left( S19 \right) \end{aligned}$$

The resonance shift and linewidth broadening are obtained similarly to Eq. (S5),

$$\begin{aligned} \delta\omega_{c,P}-i\delta\Gamma_{c,P}=\frac{g^{2}}{\Delta+i\Gamma_{a}^{'}}\#\left( S20 \right) \end{aligned}$$

in which the additional loss $\Gamma_{a}^{'}=\Gamma_{a}+\frac{\omega_{P}}{\omega_{R}}g_{R}\left| a_{R} \right|^{2}$ is a result of stimulated Raman loss (SRL). Consider the stationary approximation of $dc_{P}/dt$ = 0 and substitute the Eq. (S20) into it, we have

$$\begin{aligned} \left| a_{P} \right|^{2}=\frac{g^{2}}{\Delta^{2}+{\Gamma_{a}^{'}}^{2}}\frac{\epsilon_{P}^{2}}{\left( \Delta_{P}-\delta\omega_{c,P} \right)^{2}+\left( \Gamma_{c,P}+\delta\Gamma_{c,P}+\kappa_{\mathrm{in},P} \right)}\#\left( S21 \right) \end{aligned}$$

Similarly, with $\Gamma_{a}^{''}=\Gamma_{a}-g_{R}\left| a_{P} \right|^{2}$ (stimulated Raman gain, SRG), we obtain

$$\begin{aligned} \left| a_{R} \right|^{2}=\frac{g^{2}}{\Delta^{2}+{\Gamma_{a}^{''}}^{2}}\frac{\epsilon_{R}^{2}}{\left( \Delta_{R}-\delta\omega_{c,R} \right)^{2}+\left( \Gamma_{c,R}+\delta\Gamma_{c,R}+\kappa_{\mathrm{in},R} \right)}\#\left( S22 \right) \end{aligned}$$

The form of the Eqs. (S21) and (S22), consistent with (S9.2), imply the WGM can enhance both pump and Stokes lights for two orders of magnitude beyond nanoplasmonics, which is an experimental result of enhanced Raman spectroscopy using the WGM microprobe configuration. Furthermore, under the approximation $\frac{\omega_{P}}{\omega_{R}}g_{R}\left| a_{R} \right|^{2}\ll\Gamma_{a}$, we find that

$$\begin{aligned} \left| a_{P} \right|^{2}\approx\frac{g^{2}}{\Delta^{2}+\Gamma_{a}^{2}}\left( 1-\frac{\omega_{P}}{\omega_{R}}\frac{2\Gamma_{a}g_{R}\left| a_{R} \right|^{2}}{\Delta^{2}+\Gamma_{a}^{2}} \right)\frac{\epsilon_{P}^{2}}{\left( \Delta_{P}-\delta\omega_{c,P} \right)^{2}+\left( \Gamma_{c,P}+\delta\Gamma_{c,P}+\kappa_{\mathrm{in},P} \right)}\#\left( S23 \right) \end{aligned}$$

which indicates the pump light experiences the SRL as the Stokes light turns on ($\left| a_{R} \right|^{2}\neq0$, $g_{R}\neq0$),

$$\begin{aligned} \frac{{\Delta\left| a_{P} \right|}^{2}}{\left| a_{P} \right|^{2}}\approx\frac{\omega_{P}}{\omega_{R}}\frac{2\Gamma_{a}g_{R}}{\Delta^{2}+\Gamma_{a}^{2}}\left| a_{R} \right|^{2}\#\left( S24 \right) \end{aligned}$$

and

$$\begin{aligned} {\Delta\left| a_{P} \right|}^{2}\propto\left| a_{P} \right|^{2}\left| a_{R} \right|^{2}\#\left( S25 \right) \end{aligned}$$

The SRS signal is proportional to the intensity of both pump and Stokes lights, and therefore, the sensitivity of plasmon-enhanced SRS^10^ can be improved by 4 orders of magnitude with the WGM microprobe.


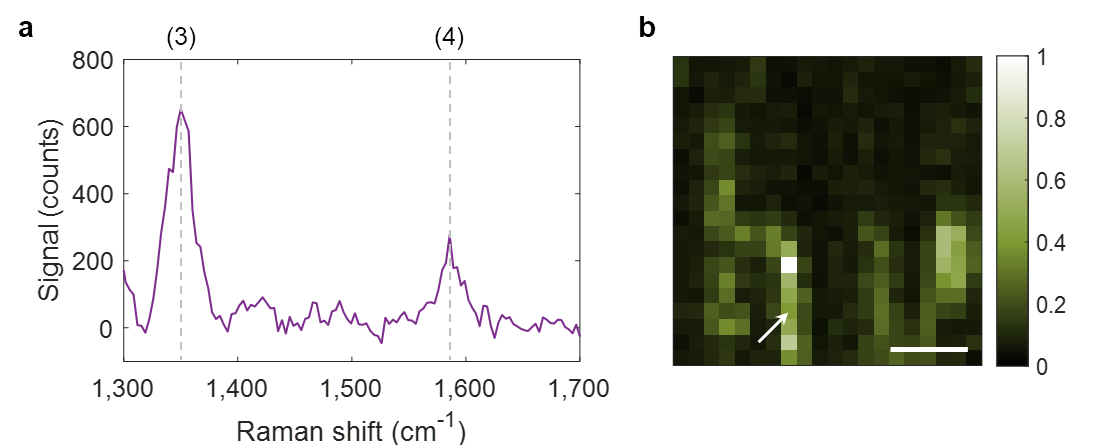


**Fig. S12 | 2D Raman imaging by tracking different vibrations.** **a**, The Raman spectra collected from the arrow-pointed pixel in **b**. **b**, Raman imaging by tracking the peak (4), generated by the stretch of the carbon ring (C-C stretch). Scale bar: 5 μm.


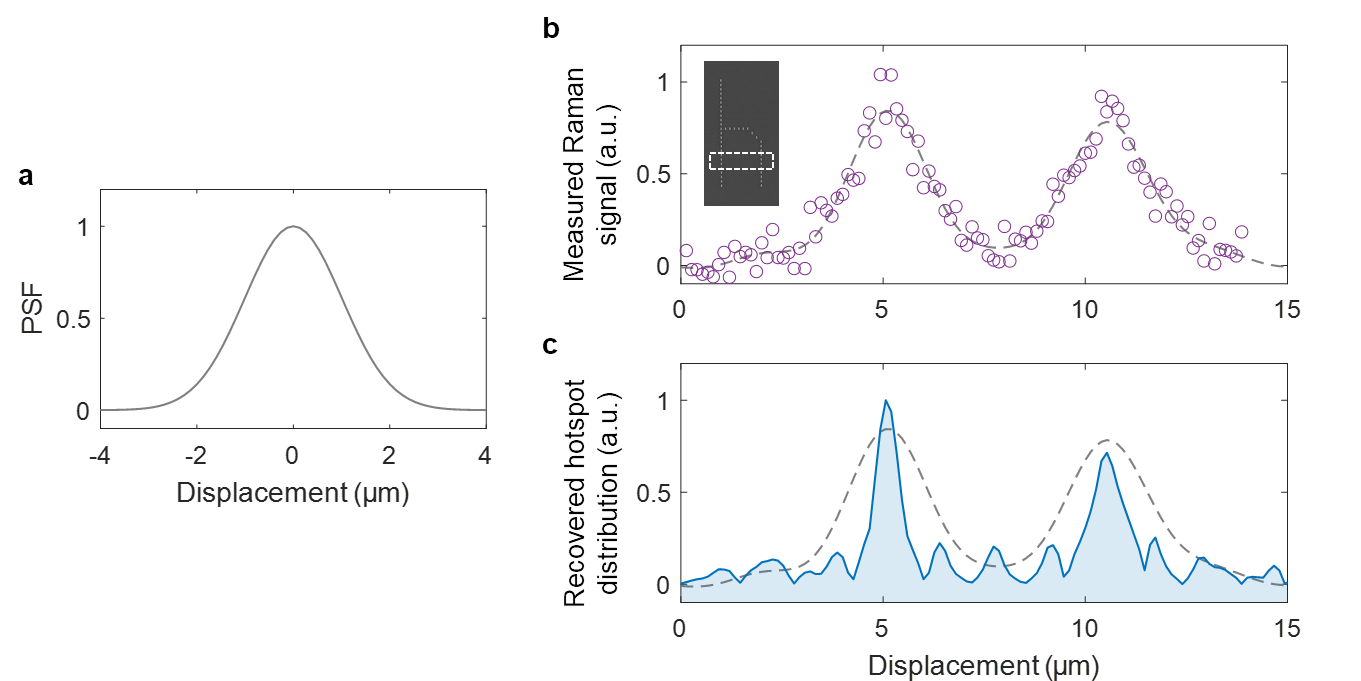


**Fig. S13 | Deconvolution algorithm for improving the imaging resolution.** **a**, Gaussian point spread function (PSF), given by the curve fitting in Fig. 4a. **b**, Measured Raman signals as scanning over two legs of the letter *h*. **c**, Recovered hotspot distribution by deconvolution algorithm. The dashed line is the reverse verification using the convolution algorithm.


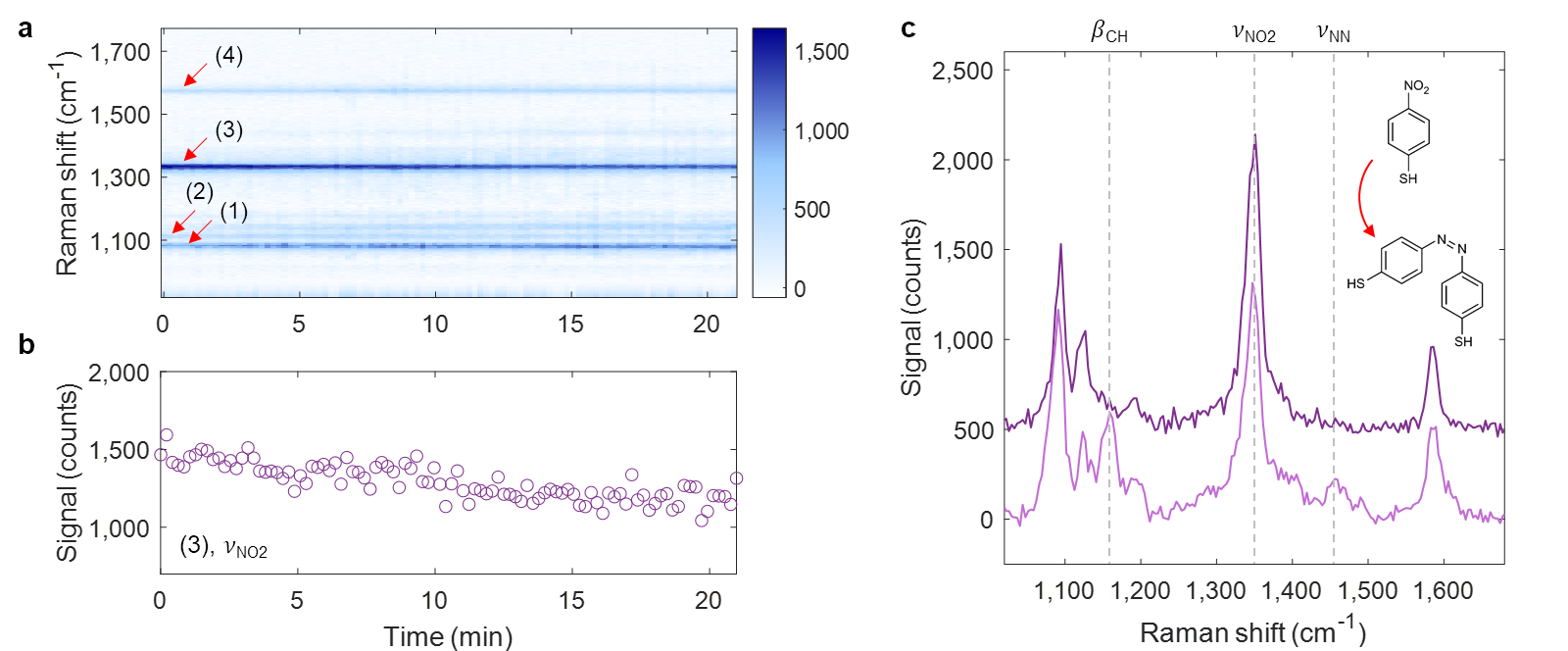


**Fig. S14 | Photocatalytic reactions at continuous optical pumping.** The nanoantennas bonded with target molecules (pNTP) are pumped by free-space light (780 nm) with a power of 2 mW. **a**, Color-coded map of time-dependent Raman spectra. (1)-(4) Raman characteristic peaks of pNTP. **b**, Decreased Raman signals of pNTP by tracking the peak (3), the symmetric stretching vibration of the NO_2_ group. **c**, The photocatalytic reduction reaction of pNTP (top) to p,p'-dimercaptoazobisbenzene (DMAB) (bottom, partially converted) after 21 min optical pumping. The peaks of DMAB-related vibrations (C-H and N=N) appear^11^.


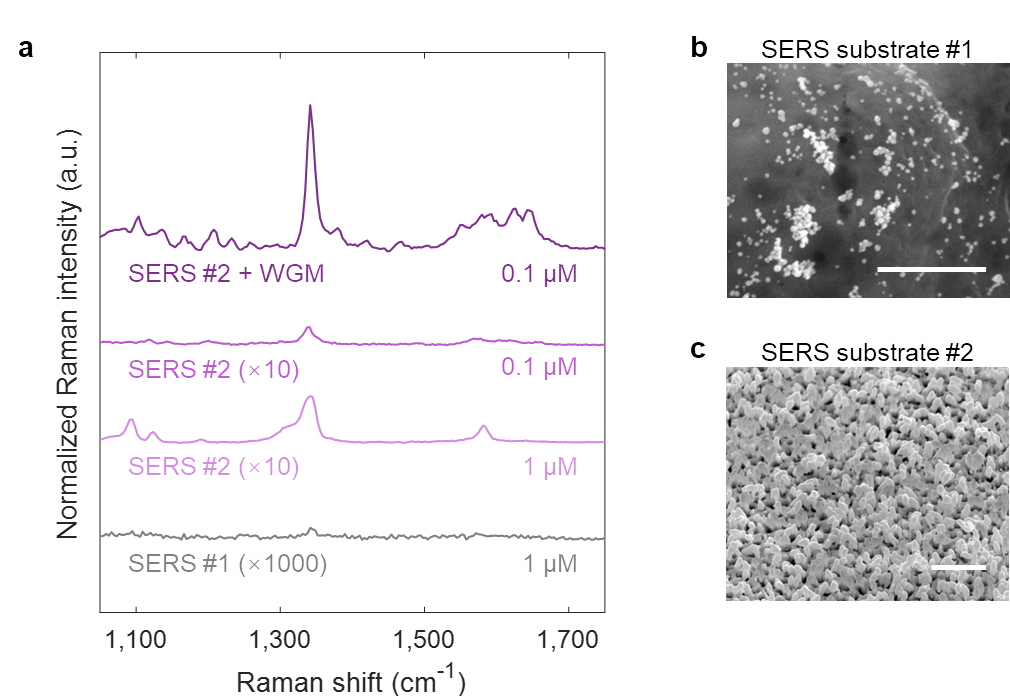


**Fig. S15 | Improvement of commercial SERS substrates by the WGM microprobe.** The potential to improve types of existing SERS substrates is demonstrated. **a**, The Raman signal of pNTP molecules (1 μM) from the free-space-pumped SERS substrate #2 was about 400 times stronger than that from the SERS substrate #1. The signal intensity decreased significantly as the molecular concentration was diluted down to 0.1 μM. Then, the Raman signal was enhanced by 80 times using the WGM microprobe in which both WGMs and SERS substrate contribute to the enhancement (the number of excited molecules were normalized as described in Methods). Pump power: 0.1 mW. Integration time: 1 s. **b**, SERS substrate #1 (StellarNet Inc.), a test paper deposited with gold nanoparticles. **c**, SERS substrate #2 (Silmeco ApS), a silicon substrate with gold nanopillars. Scale bar: 1 μm.


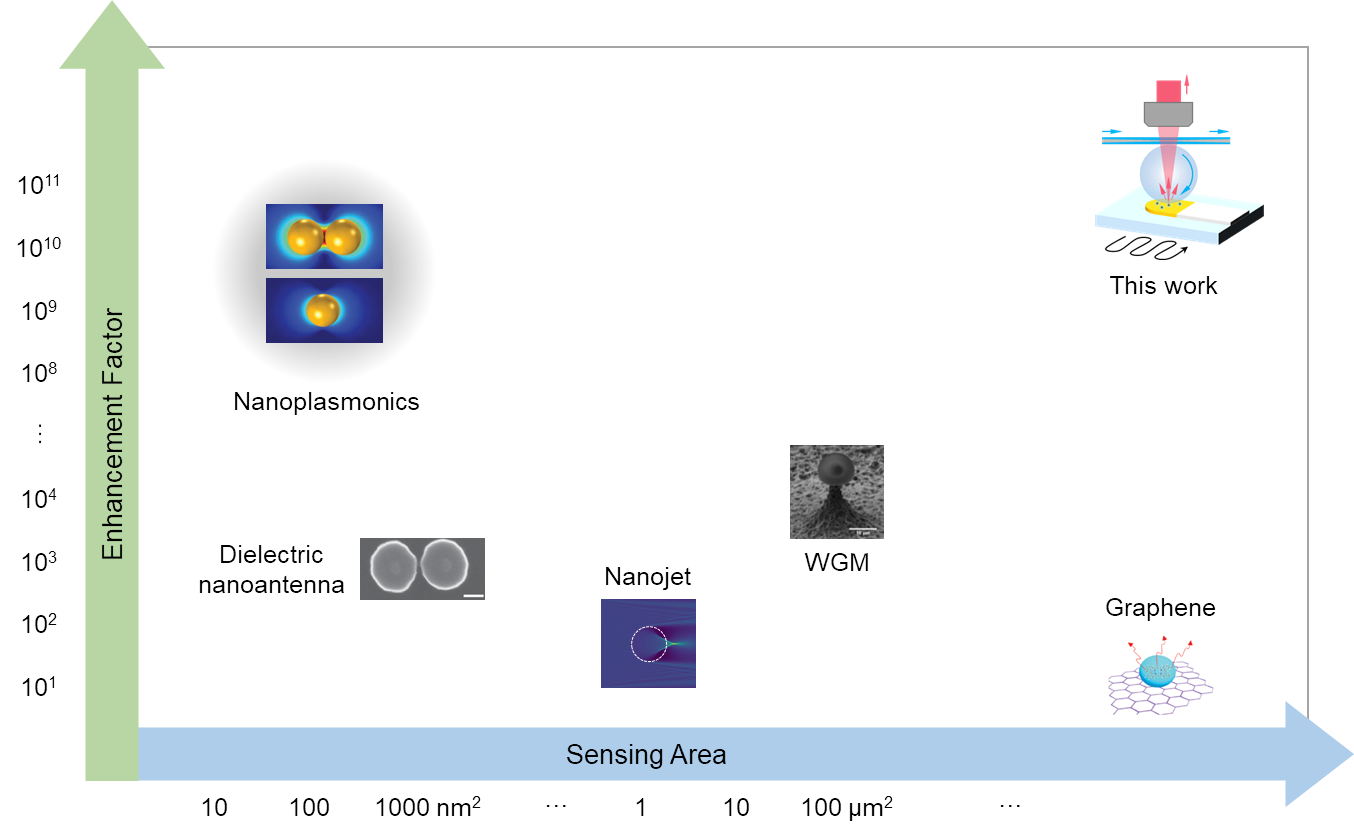


**Fig. S16 | Enhancement factor and sensing area of the structures for Raman spectroscopy.** The WGM microsphere^2^, nanoplasmonic structures^3^, nanojet^12^, silicon nanoantennas^13^, and graphene^14^ are compared. The WGM microprobe achieves much higher sensitivity for molecular detections than widely used plasmonic-based techniques as demonstrated in the main text. The sensing area can be extended to the whole sample thanks to the scanning capability of the microprobe configuration.


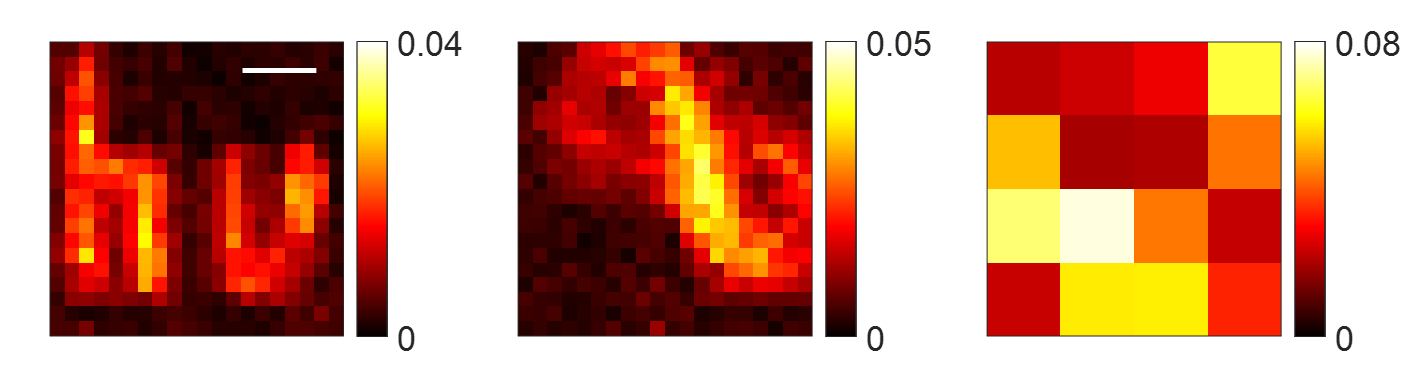


**Fig. S17 | Raman imaging acquired by free-space pump.** The intensities of each image are normalized by that acquired by the WGM microprobe (Figs. 4b-4d). The free-space imaging resolution is about two times worse than that of the WGM microprobe. Scale bar: 5 μm.

**Supplementary Reference**

1. Ruesink, F., Doeleman, H. M., Hendrikx, R., Koenderink, A. F. & Verhagen, E. Perturbing Open Cavities: Anomalous Resonance Frequency Shifts in a Hybrid Cavity-Nanoantenna System. *Phys. Rev. Lett.* **115**, 1–5 (2015).

2. Huang, S. H. *et al.* Surface-enhanced Raman scattering on dielectric microspheres with whispering gallery mode resonance. *Photonics Res.* **6**, 346 (2018).

3. Ding, S. Y. *et al.* Nanostructure-based plasmon-enhanced Raman spectroscopy for surface analysis of materials. *Nat. Rev. Mater.* **1**, (2016).

4. Pelton, M. Modified spontaneous emission in nanophotonic structures. *Nat. Photonics* **9**, 427–435 (2015).

5. Zhu, J. *et al.* On-chip single nanoparticle detection and sizing by mode splitting in an ultrahigh-Q microresonator. *Nat. Photonics* **4**, 46–49 (2010).

6. Baaske, M. D. & Vollmer, F. Optical observation of single atomic ions interacting with plasmonic nanorods in aqueous solution. *Nat. Photonics* **10**, 733–739 (2016).

7. Baaske, M. D., Foreman, M. R. & Vollmer, F. Single-molecule nucleic acid interactions monitored on a label-free microcavity biosensor platform. *Nat. Nanotechnol.* **9**, 933–939 (2014).

8. Dantham, V. R. *et al.* Label-free detection of single protein using a nanoplasmonic-photonic hybrid microcavity. *Nano Lett.* **13**, 3347–3351 (2013).

9. Yu, X. C. *et al.* Single-molecule optofluidic microsensor with interface whispering gallery modes. *Proc. Natl. Acad. Sci. U. S. A.* **119**, (2022).

10. Zong, C. *et al.* Plasmon-enhanced stimulated Raman scattering microscopy with single-molecule detection sensitivity. *Nat. Commun.* **10**, 1–11 (2019).

11. Van Schrojenstein Lantman, E. M., Deckert-Gaudig, T., Mank, A. J. G., Deckert, V. & Weckhuysen, B. M. Catalytic processes monitored at the nanoscale with tip-enhanced Raman spectroscopy. *Nat. Nanotechnol.* **7**, 583–586 (2012).

12. Dantham, V. R., Bisht, P. B. & Namboodiri, C. K. R. Enhancement of Raman scattering by two orders of magnitude using photonic nanojet of a microsphere. *J. Appl. Phys.* **109**, (2011).

13. Caldarola, M. *et al.* Non-plasmonic nanoantennas for surface enhanced spectroscopies with ultra-low heat conversion. *Nat. Commun.* **6**, (2015).

14. Ling, X. *et al.* Can graphene be used as a substrate for Raman enhancement? *Nano Lett.* **10**, 553–561 (2010).
